# Long‐read nanopore DNA sequencing can resolve complex intragenic duplication/deletion variants, providing information to enable preimplantation genetic diagnosis

**DOI:** 10.1002/pd.6089

**Published:** 2022-01-18

**Authors:** Christopher M. Watson, Deborah L. Holliday, Laura A. Crinnion, David T. Bonthron

**Affiliations:** ^1^ North East and Yorkshire Genomics Laboratory Hub Central Laboratory St. James's University Hospital Leeds UK; ^2^ Leeds Institute of Medical Research University of Leeds St. James's University Hospital Leeds UK; ^3^ Department of Clinical Genetics Leeds Teaching Hospitals NHS Trust Chapel Allerton Hospital Leeds West Yorkshire UK

## Abstract

**Background:**

The adoption of massively parallel short‐read DNA sequencing methods has greatly expanded the scope and availability of genetic testing for inherited diseases. Indeed, the power of these methods has encouraged the integration of whole genome sequencing, the most comprehensive single approach to genomic analysis, into clinical practice. Despite these advances, diagnostic techniques that incompletely resolve the precise molecular boundaries of pathogenic sequence variants continue to be routinely deployed. This can present a barrier for certain prenatal diagnostic approaches. For example, the pre‐referral workup for couples seeking preimplantation genetic diagnosis requires intragenic dosage variants to be characterised at nucleotide resolution.

**Objective:**

We sought to assess the use of long‐read nanopore sequencing to rapidly characterise an apparent heterozygous *RB1* exon 23 deletion that was initially identified by multiplex ligation‐dependent probe amplification (MLPA), in a patient with bilateral retinoblastoma.

**Methods:**

Target enrichment was performed by long‐range polymerase chain reaction (PCR) amplification prior to Flongle sequencing on a MinION long‐read sequencer.

**Results:**

Characterisation of the deletion breakpoint included an unexpected 85‐bp insertion which duplicated *RB1* exon 24 (and was undetected by MLPA). The long‐read sequence permitted design of a multiplex PCR assay, which confirmed that the mutation arose de novo.

**Conclusion:**

Our experience demonstrates the diagnostic utility of long‐read technology for the precise characterisation of structural variants, and highlights how this technology can be efficiently deployed to enable onward referral to reproductive medicine services.

## Key points

What's already known about this topic?

- Molecular diagnostic techniques that incompletely resolve pathogenic sequence variants can present a barrier for certain prenatal diagnostic approaches.

What does this study add?

- This study demonstrates how nanopore‐based sequencing could be rapidly deployed for follow‐up analysis of previously identified, but incompletely‐defined structural variants, enabling onward referral to a national preimplantation genetic diagnosis service.

## 1 INTRODUCTION

Approximately 40% of cases of retinoblastoma occur bilaterally, a finding which is indicative of heritable autosomal dominant susceptibility, attributable to a germline loss‐of‐function mutation in the *RB1* gene (OMIM: 180200). Since individuals with heritable retinoblastoma are also at increased risk of developing non‐ocular tumours, establishing a molecular diagnosis and evaluating at‐risk family members are of critical importance.

Sequence‐based analyses of the *RB1* gene, traditionally performed by Sanger sequencing of the coding exons and immediate flanking regions, identify single‐nucleotide or small insertion‐deletion variants that account for the majority (80%–85%) of pathogenic variants.^1^ The remaining heritable variants are typically either intragenic deletion/duplication events (discovered using quantitative polymerase chain reaction [PCR] or multiplex ligation‐dependent probe amplification [MLPA^®^]), or larger deletions that span the 13q14 locus (detected by chromosomal microarray). This latter group of patients may also show additional developmental delay and birth defects.^2^

A common feature of the techniques that are routinely deployed to identify dosage variants is their inability to determine the precise genomic boundaries and (in the case of copy number gains) the orientation, of the molecular event. Although the approximate minimum and maximum size of copy number variants can be estimated, the resolution is variable, depending on assay factors such as the density and locations of adjacent probes.

Over the last decade or more, the molecular diagnosis of rare genetic disorders has been revolutionized by massively parallel “next‐generation” sequencing (NGS) methods. In particular, hybridisation‐based target enrichment, combined with short‐read sequencing, has become a predominant molecular diagnostic approach. Not only single nucleotide or small insertion‐deletion variants, but also whole exon deletion or duplication events, can be identified from a single such dataset, although the detection of the latter copy‐number variants requires the use of different informatics pipelines. This has expanded the scope of molecular investigations to genes that were not typically targeted by “off‐the‐shelf” reagents, and has extended the mutation spectrum of many rare disorders.^3^ While assay sensitivity for these comparative read‐depth approaches is affected by both the underlying genomic architecture of the targeted locus, and the mean depth of sequencing for a given sample, there has nevertheless been considerable enthusiasm to incorporate these methodologies into clinical practice.^4^

Most recently, it has become feasible to deploy whole genome sequencing (WGS) for diagnostic purposes, offering greater opportunities to directly characterise structural variants at nucleotide resolution. However short‐read sequencing technologies have limited capabilities for this purpose, largely because of their inability to generate unambiguous alignments spanning low‐complexity repeat elements. Such repetitive regions are frequent sites of the breakpoints for deletions and duplications that arise due to non‐allelic homologous recombination. In addition, WGS remains an expensive diagnostic approach when a sequence variant is already partially defined. By contrast, “third‐generation” single molecule sequencers can generate long sequence reads that unambiguously define structural variants by spanning low‐complexity regions. These instruments have therefore been used for both the targeted follow‐up of complex alleles^5^, ^6^ and structural variant discovery.^7^

Here, we describe the use of a low‐throughput long‐read nanopore device, the “Flongle”, to delineate the molecular breakpoint of an apparent heterozygous *RB1* exon 23 deletion, at nucleotide resolution. We assess the accuracy of the nanopore platform and highlight the importance of retrospectively characterising incompletely defined sequence variants. Analysis of the presented case enabled onward referral to a national preimplantation genetic diagnosis service; such scenarios are likely to be of increasing clinical importance.

## 2 MATERIALS AND METHODS

An 8‐month old male infant presenting with bilateral retinoblastoma was referred for molecular genetic analysis of the *RB1* gene. Following written consent, DNA was isolated from peripheral blood lymphocytes of the proband and his relatives using the Puregene standard salting out procedure (Qiagen GmbH). Ethical approval for this study was granted by the Leeds East Research Ethics Committee (18/YH/0070).

Investigation of *RB1* by MLPA (probe mix P047; MRC Holland, Amsterdam, Netherlands), was conducted by an external laboratory following manufacturer's protocols.^8^

To more closely delineate the heterozygous *RB1* exon 23 deletion, a long‐range PCR amplicon (11,785bp) was optimised for Flongle sequencing on a MinION long‐read sequencer (Oxford Nanopore Technologies [ONT], Oxford, UK). The PCR reaction consisted of 0.5 μL of genomic DNA (210 ng/μL), 13.74 μL of nuclease‐free H_2_O, 2 μL 10× SequalPrep^TM^ reaction buffer (Invitrogen), 0.36 μL of 10× SequalPrep^TM^ 5U/μL long polymerase (Invitrogen), 0.4 μL of dimethyl sulfoxide (Invitrogen), 1 μL of 10× SequalPrep^TM^ Enhancer A (Invitrogen), and 1 μL each of 10 pmol/μL forward (dTCTGGCCCTTTGATTCCCAT) and reverse (dTGCAAGTCCTGGTTCCTCAT) primers. Oligonucleotides were purchased from Integrated DNA Technologies (Leuven). Thermocycling conditions comprised a 2 min denaturation step at 94°C; followed by 10 cycles at 94°C for 10 s, 60°C for 30 s and 68°C for 12 min; then 25 cycles at 94°C for 10 s, 60°C for 30 s and 68°C for 12 min, with an additional 20 s extension time added per cycle, before a final extension step at 72°C for 5 min. PCR products were resolved on a 1% Tris‐borate‐EDTA agarose gel then gel excised and purified using a QIAquick column (Qiagen GmbH).

To create a nanopore‐compatible sequencing library, 500 ng (68.6 fmol) of purified amplification products were first nickase/end‐repair treated. The reaction comprised 1.75 μL Ultra^TM^ II end prep reaction buffer (New England Biolabs [NEB]), 1.75 μL formalin‐fixed paraffin‐embedded (FFPE) DNA repair buffer (NEB), 1.5 μL Ultra^TM^ II end prep enzyme mix (NEB), 1.0 μL FFPE DNA repair mix (NEB), 5.9 μL PCR product and nuclease‐free water to make a total volume of 24 μL. The reaction was incubated at 20°C for 5 min then 65°C for 5 min. An AMPure XP bead clean‐up was performed (Beckman Coulter) before sequencing adaptors were ligated to the double‐stranded DNA. The reaction comprised 30 μL of PCR products, 12.5 μL of Ligation Buffer (LNB; ONT), 5.0 μL of Quick Ligase (NEB) and 2.5 μL of Adaptor Mix (ONT). The reaction was incubated at room temperature for 10 min before a further AMPure XP bead clean‐up was performed. Long Fragment Buffer (ONT) was used to wash the beads before the sample was eluted in 7 μL of Elution Buffer (ONT). A Flongle flowcell was prepared for sequencing by loading 120 μL of flowcell priming mix (3 μL of Flush Tether [ONT]) combined with 117 μL of Flush Buffer (ONT) through the priming port. 20 fmol of library (6 μL) was added to 15 μL of Sequencing Buffer (ONT) and 10 μL of Loading Beads (ONT), then loaded into the flowcell. A 24 h Flongle sequencing run was initiated using MinKNOW software v.3.6.5 (ONT).

Offline basecalling was performed using Guppy v.3.6.0 (ONT), in high accuracy calling mode, to convert raw data from fast5 to FASTQ format (http://nanoporetech.com); these data are available from the European Nucleotide Archive using study accession number PRJEB48093. Adaptor sequences were trimmed from the resulting reads using Porechop v.0.2.3 (https://github.com/rrwick/Porechop). NanoFilt v.2.20 was used to filter low quality reads (*Q* < 10) and select those within a 7500−8000 bp size range (https://github.com/wdecoster/nanofilt).^9^ Processed reads were aligned to an indexed human reference genome (build hg19) using minimap2 v.2.16. (https://github.com/lh3/minimap2).^10^ SAM‐to‐BAM file conversion, BAM file indexing and read sorting by genomic coordinate were performed using samtools v.1.9 (http://www.htslib.org/).^11^ In view of the excessive read‐depth generated by the full dataset, the resulting BAM file was downsampled to 10% of the total read count (samtools v.1.9). A consensus de novo assembly of the variant‐containing allele was generated from all available sequence reads using Canu v.2.1.1 (https://github.com/marbl/canu/).^12^ This was analysed by pairwise comparison to the human reference genome, defined by the long‐range PCR amplicon, using the Needleman‐Wunsch algorithm (https://www.ebi.ac.uk/Tools/psa/emboss_needle/).^13^ BLAST‐like alignment tool (BLAT) was used to determine the genomic coordinates of the inserted sequence (http://genome.ucsc.edu/cgi‐bin/hgBlat).^14^ Summary sequencing metrics were calculated using NanoStat v.1.1.2 (https://github.com/wdecoster/nanofilt)^9^ and aligned reads were visualised using the Integrative Genome Viewer v.2.4.10 (http://software.broadinstitute.org/software/igv/).^15^

A PCR amplicon was optimised to amplify across the deletion breakpoint. The primers used to amplify the variant allele were dTCCTCAGACATTCAAACGTGT (common forward) and dCTGGCCAGAACTTCCAACAC (variant reverse) which generated a 564‐bp PCR product. A second reverse primer dAAGGGCTTCGAGGAATGTGA (normal reverse) was designed to work in combination with the common forward primer to amplify a smaller 257 bp PCR product specific to the normal allele. Each PCR comprised 0.5 μL genomic DNA (∼200 ng/μL), 19.1 μL Megamix (Microzone Ltd., Haywards Heath, UK), 0.2 μL of 10 μM common forward primer and 0.2 μL of 10 μM reverse primer. Thermocycling conditions were, 94°C for 5 min; followed by 30 cycles of 94°C for 30 s, 55°C for 1 min and 72°C for 45 s, before a final extension step at 72°C for 5 min. Amplification products were resolved on a 2% Tris‐borate‐EDTA agarose gel before being excised and purified using a QIAquick column (Qiagen GmbH). Sanger sequencing was performed on an ABI3730 to confirm the identity of both PCR amplicons; manufacturer's protocols were followed throughout (Life Technologies Ltd., Paisley, UK). Sequence chromatograms were visualised using 4Peaks software v.1.8 (https://nucleobytes.com/4peaks/index.html). To enable robust diagnostic testing, the amplicons were optimised to work as a multiplex PCR comprising all three primers. Compared to the simplex reaction, the volume of reverse primer was halved, but all other reaction components and thermocycling conditions remained the same. Multiplex PCR products were resolved on a 2% Tris‐borate‐EDTA agarose gel.

## 3 RESULTS

The proband was referred for molecular diagnostic investigation of the *RB1* gene, having presented at 8 months old with bilateral retinoblastoma; at age 12 years he also developed an osteosarcoma of the right tibia. He had no notable family history. Initial dosage analysis by MLPA revealed a heterozygous deletion of *RB1* exon 23 (NM_000321.3), that was predicted to be out‐of‐frame, creating a frameshift in the translated RB1 protein (The probe hybridisation region was sequenced to exclude the possibility that a polymorphic sequence variant disrupted binding).

**FIGURE 1:**
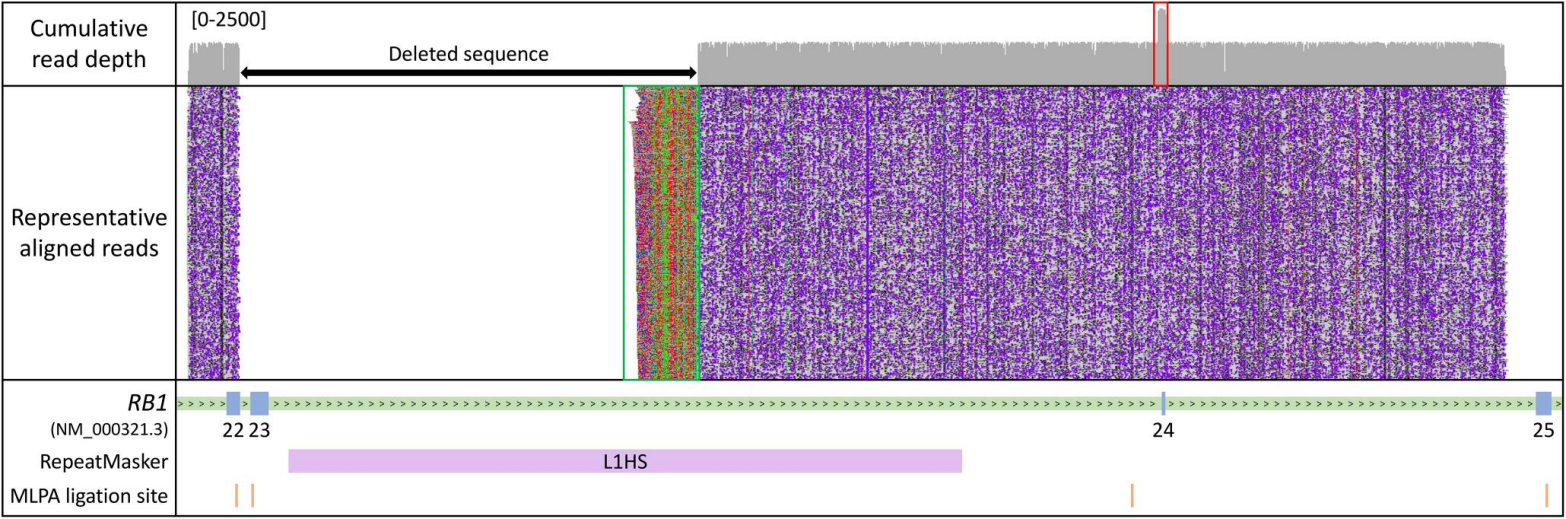
Representative long‐read sequencing alignments following long‐range polymerase chain reaction enrichment of the *RB1* target locus. Variant‐containing reads were size selected, in silico, to aid identification of the deletion breakpoint, which intersects an L1HS long interspersed nuclear element at its 3′ end. “Soft‐clipping” of the reads indicates the presence of an 85 bp insertion (see green box which corresponds to the non‐aligning portion of the read), for which 81 bp correspond to the downstream exon 24 locus (see red box which corresponds to increased read‐depth). Multiplex ligation‐dependent probe amplification (MLPA) ligation sites are marked. It is notable that the exon 24 probe is sited 265‐bp upstream of the exon boundary (and outside the duplicated segment). The *y*‐axis scale for the cumulative read‐depth plot is labelled. Arrows denote the direction of transcription

To verify and more closely delineate the exon 23 deletion, a long‐range PCR amplicon encompassing adjacent MLPA probes, was optimised (Supplementary Figure 1) and analysed by long‐read nanopore sequencing. Summary run metrics are included in Supplementary Table 1. To select reads specific to the variant‐containing allele, adaptor‐trimmed sequences were filtered by read length. Visual inspection of these data, following alignment to the human reference genome, revealed the boundaries of the deleted sequence (Figure 1). The downstream breakpoint intersects an L1HS low‐complexity long interspersed nuclear element (LINE). Further scrutiny of the downstream breakpoint revealed an abundance of “soft‐clipped” nucleotides (a portion of the read that does not align to the reference sequence); these, in combination with the increased cumulative read coverage surrounding exon 24, prompted us to perform a de novo assembly of the quality‐ and length‐filtered reads. Pairwise alignment of the resulting consensus sequence with a reference sequence spanning the amplified locus revealed the presence of an 85 bp insertion at the deletion breakpoint (Supplementary Figure 2). BLAT alignment revealed that a stretch of 81 of these 85 nucleotides perfectly matched the sequence of *RB1* exon 24, with the new sequence inserted in the sense (5′–3′) orientation (An additional four nucleotides not matching exon 24 were present at the 3′ end of the insertion.) It is noteworthy that the ligation site for the designated *RB1* “exon 24” MLPA probe is actually located 265 bp upstream of the exon 24 boundary, explaining why no alteration of exon 24 dosage had been observed.

**FIGURE 2:**
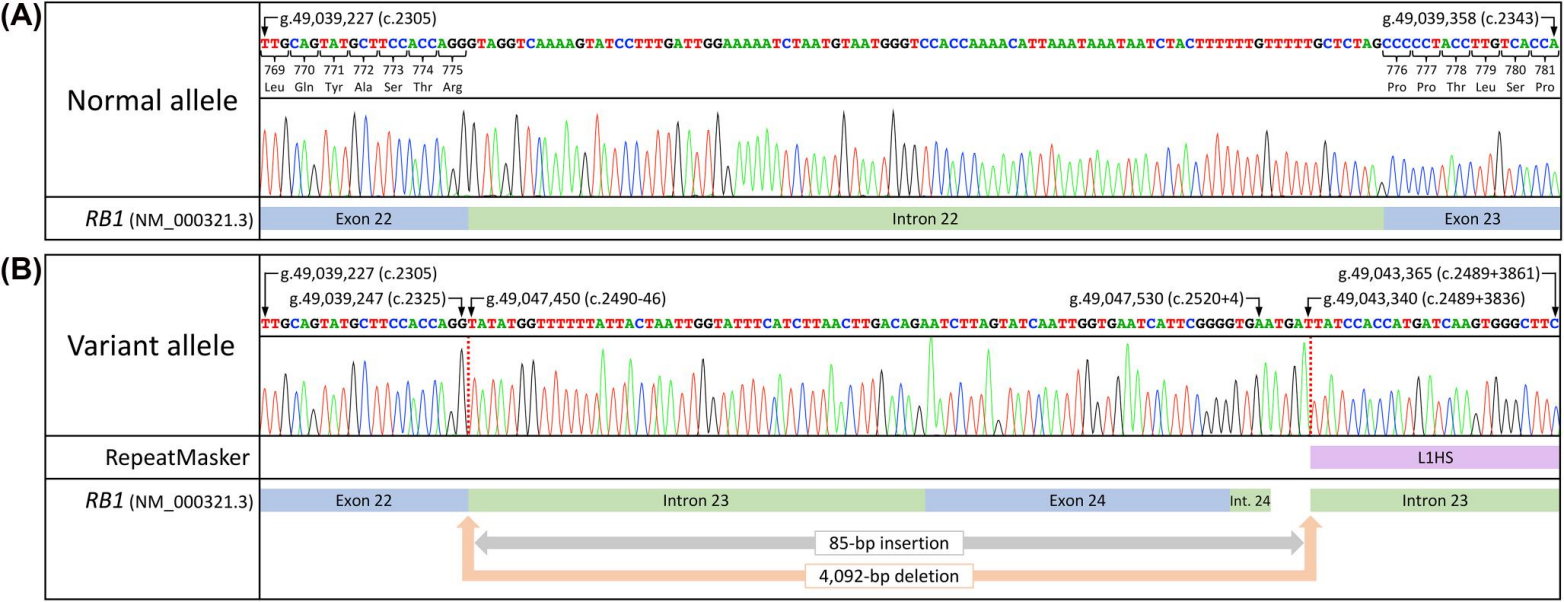
Sanger sequencing chromatograms verifying the (A) normal and (B) variant‐containing alleles. An 85 bp insertion was confirmed at the deletion breakpoint, which intersects an L1HS long interspersed nuclear element at its 3′ end. The inserted sequence encompassed the downstream exon 24 locus. Genomic coordinates are reported using human genome build hg19. Transcript numbering is according sequence NM_000321.3. Int.: Intron

To validate the variant‐containing allele, a breakpoint‐spanning PCR assay was designed. Sanger sequencing of the resulting amplification products confirmed the breakpoint, and the sequence of the insertion (Figure 2). The clinical significance of the completely characterised variant, NC_000013.10(NM_000321.3):c.2325+1_2489+3835delins (2490‐46_2520+4;ATGA), was assigned as “pathogenic” following interpretation according to the Association for Clinical Genomic Science best practice guidelines.^16^ The variant was not identified on in‐house or locus‐specific databases (http://RB1.variome.org).

To appraise the value of individual raw nanopore reads, we extracted those with the highest mean basecall quality score. Pairwise alignment between these individual reads and a Sanger‐verified curated reference, which included the variant site and adjacent sequence, yielded a maximum identity score of 98.6% (Supplementary Table 2). In addition to the mutation we describe, 23 pairwise mismatches were identified between the Canu assembly and hg19 reference sequence. Of these, 13 mismatches were located in a poly(N) region and 10 were not. Twenty‐one mismatches were Sanger‐validated; at 10 locations, the Canu‐generated assembly was correct (Supplementary Figure 3). The remaining 11 mismatch sites were all located within poly(N) tracts (that varied in length between three and eight nucleotides) and their reported lengths were incorrectly underestimated, by a single nucleotide, by the Canu‐generated assembly.

**FIGURE 3:**
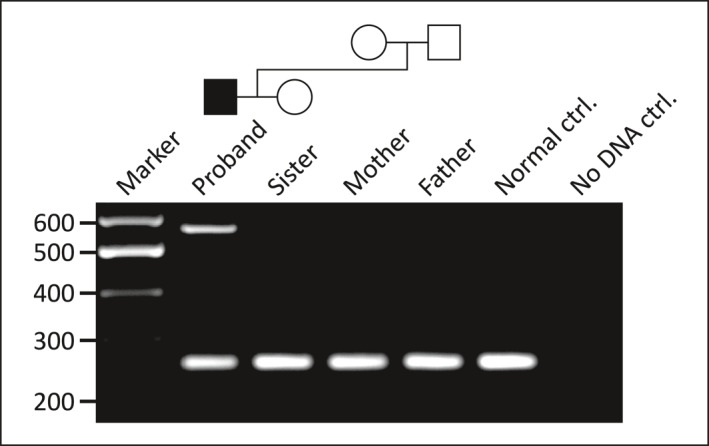
A diagnostic multiplex polymerase chain reaction (PCR) assay showing amplification products for normal (257 bp) and variant (564 bp) containing alleles. The proband is heterozygous for the deletion‐insertion allele which has arisen de novo (i.e. is absent in his parents). The variant allele was not detected in his unaffected sister

A multiplex PCR was subsequently optimised, incorporating a normal allele‐specific reverse primer to work in conjunction with the variant allele‐specific reverse primer; this provides a single assay for genotyping the indel in at‐risk individuals. It was demonstrated that the variant had arisen de novo in the proband (is absent in his parents), and the mutation was also not detected in his sister (Figure 3).

## 4 DISCUSSION

The adoption of WGS into routine clinical practice is enabling the complete characterisation of some structural variants, at nucleotide resolution, from a single assay.^17^ However, despite the continued falling cost of WGS, there remains a need to develop workflows for the retrospective characterisation of variants that have already been identified using other methods. Our approach contrasts with recent reports of prenatal investigations, using long‐read methods, in which investigators were able to identify balanced chromosomal rearrangements, directly from biopsied embryos (albeit with the assistance of whole genome amplification).^18^ Such methods have more recently been extended to enable the concurrent identification of markers, linked to the pathogenic variant, that can then be used in traditional haplotype phasing assays of embryo biopsies.^19^

To address the need for a flexible, yet widely applicable and affordable approach to the identified diagnostic need, we investigated the utility of targeted nanopore long‐read sequencing. In the case we report here, an *RB1* single exon deletion had been first identified by MLPA. Detailed molecular characterisation of the deletion was required to allow onward referral of this patient for preimplantation genetic diagnosis, because MLPA is not suitable for diagnostic analysis of embryo biopsies. Furthermore, since the identified mutation is de novo in origin, defining a high‐risk haplotype linked to the deletion, by analysis of the patient's parents, was not possible. Hence it was necessary to fully characterize the deletion so as to allow a direct molecular test.

We defined the deletion breakpoint and revealed an associated 85 bp insertion (which included exon 24) that had been overlooked by MLPA. This was due to the designated exon 24 MLPA probe hybridisation site actually being located 265 bp upstream from the 5′ exon 24 boundary. Although the constraints of MLPA probe design may preclude optimal placement of all probes, our observation does highlight a limitation of some commercial “off‐the‐shelf” reagents for identifying clinically significant intragenic deletions and duplications.

Characterisation of sequence variants at nucleotide resolution removes ambiguity and enables precise reporting in accordance with HGVS‐nomenclature.^20^ This facilitates communication between diagnostic laboratories and enables accurate curation of human variation, including the submission of variants to disease‐specific databases. Furthermore, the consensus interpretation of clinically relevant variants, through international collaborative resources such as the ClinGen initiative, is greatly improved.^21^ Resolving the precise breakpoint, of an incompletely defined variant, can aid clinical interpretation. This was previously demonstrated in a family harbouring an intragenic *CNTNAP2* deletion. While the minimum extent of the arrayCGH defined variant included intron three, short‐read sequencing confirmed that the deletion encompassed the upstream exon (located within the arrayCGH coordinates defining the variant's maximum extent) predicting an out‐of‐frame *CNTNAP2* transcript.^22^

The downstream breakpoint of the present 4 kb deletion intersects an L1HS LINE element. Nevertheless, the alignment of long‐read sequences, which are anchored by flanking, non‐repetitive and highly specific sequences, resulted in a uniform read‐depth profile. In contrast, an equivalent short‐read NGS dataset would have exhibited non‐uniform coverage, correlated with reduced mapping quality scores in regions of low sequence complexity. For deletion‐insertion variants, the likelihood of being able to span the insertion, using short reads, depends on the length of the insertion. For mobile element insertions (the ends of which contain a poly[A] tract) the magnitude of this challenge is increased, as we have previously described.^23^

To simplify the task of correctly assembling the sequences of both alleles from a mixed pool of normal and variant‐containing reads, in silico size selection was performed. We next undertook de novo assembly of the quality‐filtered read set (to overcome the increased error rate that is intrinsic to nanopore generated sequence reads) to produce a single consensus sequence for interrogation. Pairwise comparison against a curated benchmark sequence (generated following Sanger sequencing), confirmed complete concordance between these sequences, verifying the validity of our long‐read de novo assembly pipeline. We note that the lengths of poly(N) tracts were systematically underestimated, by a single nucleotide. The accuracy of the assembly is therefore likely to be dependent on the genomic architecture of the sequenced region. From an end‐user perspective, the interpretation of a single consensus sequence, in combination with BLAT, proved much simpler than visual inspection of a Sanger sequencing chromatogram.

By characterising the variant breakpoint, it was possible to design a simple assay for familial diagnostic testing. This established that the variant arose de novo in the proband. The proband's unaffected sister was shown not to have inherited the variant, a clinically important finding, since the possibility of parental germline mosaicism could not be eliminated; this was estimated to be ∼2.5% prior to testing.^24^ While the variant we describe was not identified in population or disease‐specific databases, the breakpoint assay could be used to screen cohorts of *RB1*‐mutation negative retinoblastoma patients to establish the precise prevalence of the mutation.

Our experience demonstrates how nanopore‐based sequencing, using Flongle flowcells, could be routinely deployed for follow‐up analysis of previously identified, but incompletely‐defined, structural variants. Due to high sequence yield, our analysis used a down‐sampled dataset comprising 10% of the aligned reads. This suggests that the workflow could be readily adapted to allow concurrent analysis of multiple patient libraries; allowing an increase in laboratory throughput and reducing the per‐patient assay cost. Furthermore, as nanopore reads can be analysed in real‐time, there remains an ongoing possibility that runs could be terminated once sufficient data has been accumulated, leading to an overall reduction in test turnaround times.

In summary, we report how complete characterisation of a pathogenic dosage variant can enable onward referral to a national preimplantation genetic diagnostic service. This was efficiently achieved using a facile long‐read workflow.

## CONFLICT OF INTEREST

Dr Watson has received travel expenses to speak at an ONT organized conference.

## Supporting information

Figure S1

Figure S2

Figure S3

Table S1

Table S2

## Data Availability

The data that support the findings of this study are available from the corresponding author upon reasonable request.
