# Supplementary material for: Long‐read nanopore DNA sequencing can resolve complex intragenic duplication/deletion variants, providing information to enable preimplantation genetic diagnosis
Source: Prenat Diagn. 2022 Jan 18;42(2):226–32. doi: 10.1002/pd.6089 (PMC9305782; doi:10.1002/pd.6089)
Supplement: Supplementary file 1 — Figure S1 [file PD-42-226-s004.pdf]

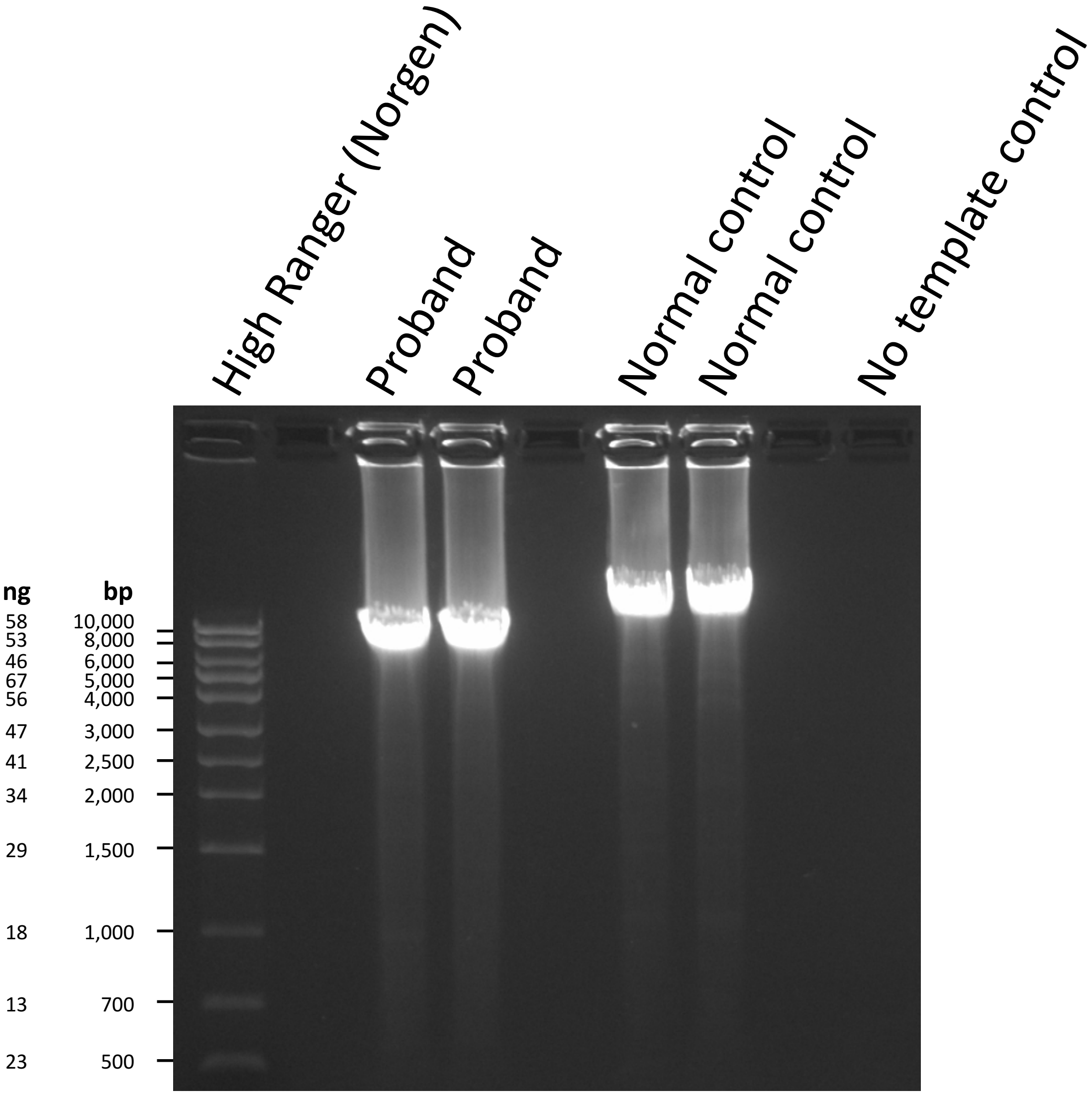

Supplementary Figure 1: Long-range PCR amplification products showing preferential amplification of the variant-containing allele. Bands were gel excised prior to QIAquick column purification and nanopore sequencing.
