# Supplementary material for: Long‐read nanopore DNA sequencing can resolve complex intragenic duplication/deletion variants, providing information to enable preimplantation genetic diagnosis
Source: Prenat Diagn. 2022 Jan 18;42(2):226–32. doi: 10.1002/pd.6089 (PMC9305782; doi:10.1002/pd.6089)
Supplement: Supplementary file 2 — Figure S2 [file PD-42-226-s003.docx]

**Supplementary Figure 2:** Pairwise alignment between the human reference genome locus defined by the long-range PCR assay primer sites, and the consensus assembly sequence derived from the long-read dataset. The region of poor sequence identity corresponds to the 4,092-bp deletion (nucleotides coloured blue). The 85 inserted nucleotides (green) are identified by their sporadic homology within the deleted region. Mismatched sites are numbered consecutively (excluding variant NC_000013.10(NM_000321.3):c.2325+1_2489+3835delins[2490-46_2520+4;ATGA]) and are highlighted cyan when located within a poly(N) tract or yellow otherwise. Primer sequences are highlighted grey.

########################################

# Program: needle

# Rundate: Sun 7 Feb 2021 16:30:51

# Commandline: needle

# -auto

# -stdout

# -asequence emboss_needle-I20210207-162700-0725-57629603-p2m.asequence

# -bsequence emboss_needle-I20210207-162700-0725-57629603-p2m.bsequence

# -datafile EDNAFULL

# -gapopen 10.0

# -gapextend 0.5

# -endopen 10.0

# -endextend 0.5

# -aformat3 pair

# -snucleotide1

# -snucleotide2

# Align_format: pair

# Report_file: stdout

########################################

#=======================================

#

# Aligned_sequences: 2

# 1: LR_PCR_ref

# 2: Canu_assem

# Matrix: EDNAFULL

# Gap_penalty: 10.0

# Extend_penalty: 0.5

#

# Length: 11796

# Identity: 7738/11796 (65.6%)

# Similarity: 7738/11796 (65.6%)

# Gaps: 4045/11796 (34.3%)

# Score: 36332.5

#

#

#=======================================

**LR_PCR_ref** 1 TCTGGCCCTTTGATTCCCATcatgctttccattctaccagtctatctact 50

||||||||||||||||||||||||||||||||||||||||||||||||

**Canu_assem** 1 --TGGCCCTTTGATTCCCATCATGCTTTCCATTCTACCAGTCTATCTACT 48

**LR_PCR_ref** 51 cctctcttcccagtctatatactcttctcttcttggatggccagctcttc 100

||||||||||||||||||||||||||||||||||||||||||||||||||

**Canu_assem** 49 CCTCTCTTCCCAGTCTATATACTCTTCTCTTCTTGGATGGCCAGCTCTTC 98

**LR_PCR_ref** 101 catctgctgctgcctggctatttctctcaatcattctgtgacatttcact 150

||||||||||||||||||||||||||||||||||||||||||||||||||

**Canu_assem** 99 CATCTGCTGCTGCCTGGCTATTTCTCTCAATCATTCTGTGACATTTCACT 148

**LR_PCR_ref** 151 tctagaagagcagctataatccaagcctaagaagtaattttatttattta 200

||||||||||||||||||||||||||||||||||||||||||||||||||

**Canu_assem** 149 TCTAGAAGAGCAGCTATAATCCAAGCCTAAGAAGTAATTTTATTTATTTA 198

**LR_PCR_ref** 201 ttattttttcctttataatatgtgcttcttaccagtcaaaaagtattata 250

||||||||||||||||||||||||||||||||||||||||||||||||||

**Canu_assem** 199 TTATTTTTTCCTTTATAATATGTGCTTCTTACCAGTCAAAAAGTATTATA 248

**LR_PCR_ref** 251 aactattagaaaagaaaatctaaaggtagaaattttaaaattcatttaac 300

||||||||||||||||||||||||||||||||||||||||||||||||||

**Canu_assem** 249 AACTATTAGAAAAGAAAATCTAAAGGTAGAAATTTTAAAATTCATTTAAC 298

**LR_PCR_ref** 301 aagtaaattttacttttttttttttttttttttttttactgttcttcctc 350

[1]

||||||||||||| |||||||||||||||||||||||||||

**Canu_assem** 299 AAGTAAATTTTAC----------TTTTTTTTTTTTTTACTGTTCTTCCTC 338

**LR_PCR_ref** 351 agacattcaaacgtgttttgatcaaagaagaggagtatgattctattata 400

||||||||||||||||||||||||||||||||||||||||||||||||||

**Canu_assem** 339 AGACATTCAAACGTGTTTTGATCAAAGAAGAGGAGTATGATTCTATTATA 388

**LR_PCR_ref** 401 gtattctataactcggtcttcatgcagagactgaaaacaaatattttgca 450

||||||||||||||||||||||||||||||||||||||||||||||||||

**Canu_assem** 389 GTATTCTATAACTCGGTCTTCATGCAGAGACTGAAAACAAATATTTTGCA 438

**LR_PCR_ref** 451 gtatgcttccaccagg**gtaggtcaaaagtatcctttgattggaaaaatct** 500

||||||||||||||||

**Canu_assem** 439 GTATGCTTCCACCAGG---------------------------------- 454

**LR_PCR_ref** 501 **aatgtaatgggtccaccaaaacattaaataaataatctacttttttgttt** 550

**Canu_assem** 455 -------------------------------------------------- 454

**LR_PCR_ref** 551 **ttgctctagccccctaccttgtcaccaatacctcacattcctcgaagccc** 600

**Canu_assem** 455 -------------------------------------------------- 454

**LR_PCR_ref** 601 **ttacaagtttcctagttcacccttacggattcctggagggaacatctata** 650

||||

**Canu_assem** 455 ----------------------------------------------**TATA** 458

**LR_PCR_ref** 651 **tttcacccctgaagagtccatataaaatttcagaaggtctgccaacacca** 700

|

**Canu_assem** 459 **T**------------------------------------------------- 459

**LR_PCR_ref** 701 **acaaaaatgactccaagatcaaggtgtgtgttttctctttagggaagtag** 750

**Canu_assem** 460 -------------------------------------------------- 459

**LR_PCR_ref** 751 **taaagaatgagagggggattattttgatccaagaataaaaaatataaagc** 800

**Canu_assem** 460 -------------------------------------------------- 459

**LR_PCR_ref** 801 **attcttcatttcaaataagctagactcttgaaactctatttgcttattta** 850

**Canu_assem** 460 -------------------------------------------------- 459

**LR_PCR_ref** 851 **agtaacataataagaatatgggggcggggtgaagaaaatctatttacgac** 900

**Canu_assem** 460 -------------------------------------------------- 459

**LR_PCR_ref** 901 **ttaagcaacgcaagatggccgaataggaacagctccggtctacagctccc** 950

**Canu_assem** 460 -------------------------------------------------- 459

**LR_PCR_ref** 951 **agcgtgagcgacgcagaagacgggtgatttctgcatttccatctgaggta** 1000

**Canu_assem** 460 -------------------------------------------------- 459

**LR_PCR_ref** 1001 **ccgggttcatctcactagggagtgccagacagtgggcgcaggccagtgtg** 1050

**Canu_assem** 460 -------------------------------------------------- 459

**LR_PCR_ref** 1051 **tgtgcgcaccgtgtgcgagccgaagcagggcgaggcattgcctcacctgg** 1100

**Canu_assem** 460 -------------------------------------------------- 459

**LR_PCR_ref** 1101 **gaagcgcaaggggtcagggagttccctttccgagtcaaagaaaggggtga** 1150

**Canu_assem** 460 -------------------------------------------------- 459

**LR_PCR_ref** 1151 **cggacgcacctggaaaatcgggtcactcccacccgaatattgcacttttc** 1200

**Canu_assem** 460 -------------------------------------------------- 459

**LR_PCR_ref** 1201 **agaccggcttaagaaacggcgcaccacgagactatatcccacacctggct** 1250

**Canu_assem** 460 -------------------------------------------------- 459

**LR_PCR_ref** 1251 **cagagggtcctacgcccacggaatctcgctgattgctagcacagcagtct** 1300

**Canu_assem** 460 -------------------------------------------------- 459

**LR_PCR_ref** 1301 **gtgatcaaactgcaaggcggcagcgaggctgggggaggggcgcctgccat** 1350

**Canu_assem** 460 -------------------------------------------------- 459

**LR_PCR_ref** 1351 **tgcccaggcttgcttaggtaaacaaagcagccgggaagctcgaactgggt** 1400

**Canu_assem** 460 -------------------------------------------------- 459

**LR_PCR_ref** 1401 **ggagcccaccacaggtcaaggaggcctgcctgcctttgtaggctccacct** 1450

**Canu_assem** 460 -------------------------------------------------- 459

**LR_PCR_ref** 1451 **ctgggggcagggcacagacaaacaaaaagacagcagtaacctctgcagac** 1500

**Canu_assem** 460 -------------------------------------------------- 459

**LR_PCR_ref** 1501 **ttaagtgtccctgtctgacagctttgaagagagcagtggttctcccagca** 1550

**Canu_assem** 460 -------------------------------------------------- 459

**LR_PCR_ref** 1551 **cgcagctggagatctgagaacgggcagactgcctcctcaagtgggtccct** 1600

**Canu_assem** 460 -------------------------------------------------- 459

**LR_PCR_ref** 1601 **gacccctgacccccgagcagcctaactgggaggcaccccccagcaggggc** 1650

**Canu_assem** 460 -------------------------------------------------- 459

**LR_PCR_ref** 1651 **acactgacacctcacaaggcagggtattccaacagacctgcagctgaggg** 1700

**Canu_assem** 460 -------------------------------------------------- 459

**LR_PCR_ref** 1701 **tcctgtctgttagaaggaaaactaacaaccagaaaggacatctacaccga** 1750

**Canu_assem** 460 -------------------------------------------------- 459

**LR_PCR_ref** 1751 **aaacccatctgtacatcaccatcatcaaagaccaaaagtagataaaacca** 1800

**Canu_assem** 460 -------------------------------------------------- 459

**LR_PCR_ref** 1801 **caaagatggggaaaaaacagaacagaaaaactggaaactctaaaacgcag** 1850

**Canu_assem** 460 -------------------------------------------------- 459

**LR_PCR_ref** 1851 **agcgcctctcctcctccaaaggaacacagttcctcaccagcaacggaaca** 1900

**Canu_assem** 460 -------------------------------------------------- 459

**LR_PCR_ref** 1901 **aagttggacggagaatgactttgacgagctgagagaagaaggtttcagac** 1950

|||||

**Canu_assem** 460 ----------------------------------------**GGTTT**----- 464

**LR_PCR_ref** 1951 **gatcaaattactctgagctacgggaggacattcaaaccaaaggcaaagaa** 2000

**Canu_assem** 465 -------------------------------------------------- 464

**LR_PCR_ref** 2001 **gttgaaaactttgaaaaaaatttagaagaatatataactagaataaccaa** 2050

|.|||.||||

**Canu_assem** 465 ------------------------------**TTTATTACTA**---------- 474

**LR_PCR_ref** 2051 **tacagagaagtgcttaaaggagctgatggagctgaaaaccaaggctcgag** 2100

**Canu_assem** 475 -------------------------------------------------- 474

**LR_PCR_ref** 2101 **aactacgtgaagaatgcagaagcctcaggagccaatgcgatcaactggaa** 2150

**Canu_assem** 475 -------------------------------------------------- 474

**LR_PCR_ref** 2151 **gaaagggtatcagcaatggaagatgaaatgaatgaaatgaagtgagaagg** 2200

**Canu_assem** 475 -------------------------------------------------- 474

**LR_PCR_ref** 2201 **gaagtttagagaaaaaagaataaaaagaaatgagcaaagcctccaagaaa** 2250

**Canu_assem** 475 -------------------------------------------------- 474

**LR_PCR_ref** 2251 **tatgggactatgtgaaaagaccaaatctacgtctgattggtgtacctgaa** 2300

||||||

**Canu_assem** 475 -----------------------------------**ATTGGT**--------- 480

**LR_PCR_ref** 2301 **agtgatgtggagaatggaaccaagttggaaaacactctgcaggatattat** 2350

**Canu_assem** 481 -------------------------------------------------- 480

**LR_PCR_ref** 2351 **ccaggagaacttccccaatctagcaaggcaggccaacgttcagattcagg** 2400

**Canu_assem** 481 -------------------------------------------------- 480

**LR_PCR_ref** 2401 **aaatacagagaacgccacaaagatactcctcgagaagagcaactccaaga** 2450

**Canu_assem** 481 -------------------------------------------------- 480

**LR_PCR_ref** 2451 **cacataattgtcagattcaccaaagttgaaatgaaggaaaaaatgttaag** 2500

**Canu_assem** 481 -------------------------------------------------- 480

**LR_PCR_ref** 2501 **ggcagccagagagaaaggtcgggttaccctcaaaggaaagcccatcagac** 2550

**Canu_assem** 481 -------------------------------------------------- 480

**LR_PCR_ref** 2551 **taacagcggatctctcggcagaaaccttacaagccagaagagagtggggg** 2600

**Canu_assem** 481 -------------------------------------------------- 480

**LR_PCR_ref** 2601 **ccaatattcaacattcttaaagaaaagaattttcaacccagaatttcata** 2650

|||||||

**Canu_assem** 481 ------------------------------------------**ATTTCAT**- 487

**LR_PCR_ref** 2651 **tccagccaaactaagcttcataagtgaaggagaaataaaatcctttacag** 2700

|||

**Canu_assem** 488 ---------------**CTT**-------------------------------- 490

**LR_PCR_ref** 2701 **acaagcaaatgctgagagattttgtcaccaccaggcctgccctaaaagag** 2750

**Canu_assem** 491 -------------------------------------------------- 490

**LR_PCR_ref** 2751 **ctcctgaaggaagcgctaaacatggaaaggaacaaccggtaccagccgct** 2800

**Canu_assem** 491 -------------------------------------------------- 490

**LR_PCR_ref** 2801 **gcaaaatcatgccaaaatgtaaagaccatcgagactaggaagaaactgca** 2850

||||

**Canu_assem** 491 -------------------------------------------**AACT**--- 494

**LR_PCR_ref** 2851 **tcaactaatgagcaaaatcaccagctaacatcataatgacaggatcaaat** 2900

||||||

**Canu_assem** 495 ------------------------------------**TGACAG**-------- 500

**LR_PCR_ref** 2901 **tcacacataacaatattaactttaaatataaatggactaaattctgcaat** 2950

**Canu_assem** 501 -------------------------------------------------- 500

**LR_PCR_ref** 2951 **taaaagacacagactggcaagttggataaagagtcaagacccatcagtgt** 3000

**Canu_assem** 501 -------------------------------------------------- 500

**LR_PCR_ref** 3001 **gctgtattcaggaaacccatctcacgtgcagagacacacataggctcaaa** 3050

**Canu_assem** 501 -------------------------------------------------- 500

**LR_PCR_ref** 3051 **ataaaaggatggaggaagatctaccaagccaatggaaaacaaaaaaaggc** 3100

**Canu_assem** 501 -------------------------------------------------- 500

**LR_PCR_ref** 3101 **aggggttgcaatcctagtctcggataaaacagactttaaaccaacaaaga** 3150

||||.||||

**Canu_assem** 501 ---------**AATCTTAGT**-------------------------------- 509

**LR_PCR_ref** 3151 **tcaaaagagacaaagaaggccattacataatggtaaagggatcaattcaa** 3200

|||||||

**Canu_assem** 510 ----------------------------------------**ATCAATT**--- 516

**LR_PCR_ref** 3201 **caagaggagctaactatcctaaatgtttatgcacccaatacaggagcacc** 3250

||

**Canu_assem** 517 -----**GG**------------------------------------------- 518

**LR_PCR_ref** 3251 **cagattcataaagcaagtcctgagtgacctacaaagagacttagactccc** 3300

**Canu_assem** 519 -------------------------------------------------- 518

**LR_PCR_ref** 3301 **acacattaataatgggagactttaacaccccactgtcaacattagacaga** 3350

**Canu_assem** 519 -------------------------------------------------- 518

**LR_PCR_ref** 3351 **tcaacgagacagaaagtcaacaaggatacccaggaattgaactcagctct** 3400

**Canu_assem** 519 -------------------------------------------------- 518

**LR_PCR_ref** 3401 **gcaccaagcagacctaatagacatctacagaactctccaccccaaatcaa** 3450

**Canu_assem** 519 -------------------------------------------------- 518

**LR_PCR_ref** 3451 **cagaatatacctttttttcagcaccacaccacacctattccaaaattgac** 3500

**Canu_assem** 519 -------------------------------------------------- 518

**LR_PCR_ref** 3501 **cacatagttggaagtaaagctctcctcagcaaatgtaaaagaacagaaat** 3550

**Canu_assem** 519 -------------------------------------------------- 518

**LR_PCR_ref** 3551 **tataacaaactatctctcagaccacagtgcaatcaaactagaactcagga** 3600

**Canu_assem** 519 -------------------------------------------------- 518

**LR_PCR_ref** 3601 **ttaagaatctcactcaaagccgctcaactacatggaaactgaacaacctg** 3650

**Canu_assem** 519 -------------------------------------------------- 518

**LR_PCR_ref** 3651 **ctcctgaatgactactgggtacataacgaaatgaaggcagaaataaagat** 3700

|||||

**Canu_assem** 519 ----**TGAAT**----------------------------------------- 523

**LR_PCR_ref** 3701 **gttctttgaaaccaacgagaacaaagacaccacataccagaatctctggg** 3750

**Canu_assem** 524 -------------------------------------------------- 523

**LR_PCR_ref** 3751 **acacattcaaagcagtgtgtagagggaaatttatagcactaaatgcctac** 3800

|||||

**Canu_assem** 524 ---**CATTC**------------------------------------------ 528

**LR_PCR_ref** 3801 **aagagaaagcaggaaagatccaaaattgacaccctaacatcacaattaaa** 3850

**Canu_assem** 529 -------------------------------------------------- 528

**LR_PCR_ref** 3851 **agaactagaaaagcaagagcaaacacattcaaaagctagcagaaggcaag** 3900

**Canu_assem** 529 -------------------------------------------------- 528

**LR_PCR_ref** 3901 **aaataactaaaatcagagcagaactgaaggaaatagagacacaaaaaacc** 3950

**Canu_assem** 529 -------------------------------------------------- 528

**LR_PCR_ref** 3951 **cttcaaaaaaatcaatgaatccaggagctggttttttgaaaggatcaaca** 4000

**Canu_assem** 529 -------------------------------------------------- 528

**LR_PCR_ref** 4001 **aaattgatagaccgctagcaagactaataaagaaaaaaagagagaagaat** 4050

**Canu_assem** 529 -------------------------------------------------- 528

**LR_PCR_ref** 4051 **caaatagacacaataaaaaatgataaaggggatatcaccaccgatcccac** 4100

||||

**Canu_assem** 529 ---------------------------**GGGG**------------------- 532

**LR_PCR_ref** 4101 **agaaatacaaactaccatcagagaatactacaaacacctctacgcaaata** 4150

**Canu_assem** 533 -------------------------------------------------- 532

**LR_PCR_ref** 4151 **aactagaaaatctagaggaaatggatacattcctcgacacatacactctc** 4200

**Canu_assem** 533 -------------------------------------------------- 532

**LR_PCR_ref** 4201 **ccaagactaaaccaggaagaagttgaatctctgaatagaccaataacagg** 4250

|||||

**Canu_assem** 533 -----------------------**TGAAT**---------------------- 537

**LR_PCR_ref** 4251 **ctctgaaattgtggcaataatcaatagtttaccaaccaaaaagagtccag** 4300

**Canu_assem** 538 -------------------------------------------------- 537

**LR_PCR_ref** 4301 **gaccagatggattcacagccgaattctaccagaggtacaaggaggaactg** 4350

**Canu_assem** 538 -------------------------------------------------- 537

**LR_PCR_ref** 4351 **gtaccattccttctgaaactattccaatcaatagaaaaagagggaatcct** 4400

**Canu_assem** 538 -------------------------------------------------- 537

**LR_PCR_ref** 4401 **ccctaactcattttatgaggccagcatcattctgataccaaagccgggca** 4450

**Canu_assem** 538 -------------------------------------------------- 537

**LR_PCR_ref** 4451 **gagacacagccaaaaaagagaattttagaccaatatccttgatgaacatt** 4500

**Canu_assem** 538 -------------------------------------------------- 537

**LR_PCR_ref** 4501 **gatgcaaaaatcctcaataaaatactggcaaaccgaatccagcagcacat** 4550

**Canu_assem** 538 -------------------------------------------------- 537

**LR_PCR_ref** 4551 **caaaaagc**ttatccaccatgatcaagtgggcttcatccctgggatgcaag 4600

|.||||||||||||||||||||||||||||||||||||||||||

**Canu_assem** 538 ------**GA**TTATCCACCATGATCAAGTGGGCTTCATCCCTGGGATGCAAG 581

**LR_PCR_ref** 4601 gctggttcaatatacgcaaatcaataaatgtaatccagcatataaacaga 4650

||||||||||||||||||||||||||||||||||||||||||||||||||

**Canu_assem** 582 GCTGGTTCAATATACGCAAATCAATAAATGTAATCCAGCATATAAACAGA 631

**LR_PCR_ref** 4651 gccaaagacaaaaaccacatgattatctcaatagatgcagaaaaagcctt 4700

||||||||||||||||||||||||||||||||||||||||||||||||||

**Canu_assem** 632 GCCAAAGACAAAAACCACATGATTATCTCAATAGATGCAGAAAAAGCCTT 681

**LR_PCR_ref** 4701 tgacaaaattcaacaacccttcatgctaaaaactctcaataaattaggta 4750

||||||||||||||||||||||||||||||||||||||||||||||||||

**Canu_assem** 682 TGACAAAATTCAACAACCCTTCATGCTAAAAACTCTCAATAAATTAGGTA 731

**LR_PCR_ref** 4751 ttgatgggacatatttcaaaataataagagctatctatgacaaacccaca 4800

||||||||||||||||||||||||||||||||||||||||||||||||||

**Canu_assem** 732 TTGATGGGACATATTTCAAAATAATAAGAGCTATCTATGACAAACCCACA 781

**LR_PCR_ref** 4801 gccaatatcatactgaatgggcaaaaactggaagcattccctttgaaaac 4850

||||||||||||||||||||||||||||||||||||||||||||||||||

**Canu_assem** 782 GCCAATATCATACTGAATGGGCAAAAACTGGAAGCATTCCCTTTGAAAAC 831

**LR_PCR_ref** 4851 tggcacaagacagggatgccctctctcaccgctcctattcaacatagtgt 4900

||||||||||||||||||||||||||||||||||||||||||||||||||

**Canu_assem** 832 TGGCACAAGACAGGGATGCCCTCTCTCACCGCTCCTATTCAACATAGTGT 881

**LR_PCR_ref** 4901 tggaagttctggccagggcaatcaggcaggagaaggaaataaagggtatt 4950

||||||||||||||||||||||||||||||||||||||||||||||||||

**Canu_assem** 882 TGGAAGTTCTGGCCAGGGCAATCAGGCAGGAGAAGGAAATAAAGGGTATT 931

**LR_PCR_ref** 4951 caattaggaaaagaggaagtcaaattgtccctgtttgcagatgacatgat 5000

||||||||||||||||||||||||||||||||||||||||||||||||||

**Canu_assem** 932 CAATTAGGAAAAGAGGAAGTCAAATTGTCCCTGTTTGCAGATGACATGAT 981

[2]

**LR_PCR_ref** 5001 tgtttatctagaaaaccccatcgtctcagcccaaaatctccttaagctga 5050

|||||||||||||||||||||.||||||||||||||||||||||||||||

**Canu_assem** 982 TGTTTATCTAGAAAACCCCATTGTCTCAGCCCAAAATCTCCTTAAGCTGA 1031

**LR_PCR_ref** 5051 taagcaacttcagcaaagtctcaggatacaaaatcaatgtacaaaaatca 5100

[3]

|||.||||||||||||||||||||||||||||||||||||||||||||||

**Canu_assem** 1032 TAAACAACTTCAGCAAAGTCTCAGGATACAAAATCAATGTACAAAAATCA 1081

**LR_PCR_ref** 5101 caagcattcttatacaccaacaacagacaaacagagagccaaatcatgag 5150

||||||||||||||||||||||||||||||||||||||||||||||||||

**Canu_assem** 1082 CAAGCATTCTTATACACCAACAACAGACAAACAGAGAGCCAAATCATGAG 1131

**LR_PCR_ref** 5151 tgaactcccattcacaattgcttcaaagagaataaaatacctaggaatcc 5200

||||||||||||||||||||||||||||||||||||||||||||||||||

**Canu_assem** 1132 TGAACTCCCATTCACAATTGCTTCAAAGAGAATAAAATACCTAGGAATCC 1181

**LR_PCR_ref** 5201 aacttacaagagatgtgaaggacctcttcaaggagaactacaaaccactg 5250

||||||||||||||||||||||||||||||||||||||||||||||||||

**Canu_assem** 1182 AACTTACAAGAGATGTGAAGGACCTCTTCAAGGAGAACTACAAACCACTG 1231

**LR_PCR_ref** 5251 ctcaaggaaataaaagaggacacaaacaaatggaagaacattccatgctc 5300

||||||||||||||||||||||||||||||||||||||||||||||||||

**Canu_assem** 1232 CTCAAGGAAATAAAAGAGGACACAAACAAATGGAAGAACATTCCATGCTC 1281

**LR_PCR_ref** 5301 atgggtaggaagaatcaatatcgtgaaaatggccatactgcccaaggtaa 5350

||||||||||||||||||||||||||||||||||||||||||||||||||

**Canu_assem** 1282 ATGGGTAGGAAGAATCAATATCGTGAAAATGGCCATACTGCCCAAGGTAA 1331

[4]

**LR_PCR_ref** 5351 tttacagattcgatgccatccccatcaagctaccaatgactttcttcaca 5400

|||||||||||.||||||||||||||||||||||||||||||||||||||

**Canu_assem** 1332 TTTACAGATTCAATGCCATCCCCATCAAGCTACCAATGACTTTCTTCACA 1381

**LR_PCR_ref** 5401 gaattggaaaaaactactttaaagttcatatggaaccaaaaaagagcccg 5450

||||||||||||||||||||||||||||||||||||||||||||||||||

**Canu_assem** 1382 GAATTGGAAAAAACTACTTTAAAGTTCATATGGAACCAAAAAAGAGCCCG 1431

**LR_PCR_ref** 5451 catcgccaagtcaatcctaagccaaaagaacaaagctggaggcatcacac 5500

||||||||||||||||||||||||||||||||||||||||||||||||||

**Canu_assem** 1432 CATCGCCAAGTCAATCCTAAGCCAAAAGAACAAAGCTGGAGGCATCACAC 1481

**LR_PCR_ref** 5501 tacctgacttcaaactatactacaaggctacagtaaccaaaacagcatgg 5550

||||||||||||||||||||||||||||||||||||||||||||||||||

**Canu_assem** 1482 TACCTGACTTCAAACTATACTACAAGGCTACAGTAACCAAAACAGCATGG 1531

**LR_PCR_ref** 5551 tactggtaccaaaacagagatatagatcaatggaacagaacagagccctc 5600

||||||||||||||||||||||||||||||||||||||||||||||||||

**Canu_assem** 1532 TACTGGTACCAAAACAGAGATATAGATCAATGGAACAGAACAGAGCCCTC 1581

**LR_PCR_ref** 5601 agaaataatgccgcatatctacaactatctgatctttgacaaacctgaga 5650

||||||||||||||||||||||||||||||||||||||||||||||||||

**Canu_assem** 1582 AGAAATAATGCCGCATATCTACAACTATCTGATCTTTGACAAACCTGAGA 1631

**LR_PCR_ref** 5651 aaaacaagcaatggggaaaggattccctatttaataaatggtgctgggaa 5700

||||||||||||||||||||||||||||||||||||||||||||||||||

**Canu_assem** 1632 AAAACAAGCAATGGGGAAAGGATTCCCTATTTAATAAATGGTGCTGGGAA 1681

**LR_PCR_ref** 5701 aactggctagccatatgtagaaagctgaaactggatcccttccttacacc 5750

||||||||||||||||||||||||||||||||||||||||||||||||||

**Canu_assem** 1682 AACTGGCTAGCCATATGTAGAAAGCTGAAACTGGATCCCTTCCTTACACC 1731

**LR_PCR_ref** 5751 ttatacaaaaatcaattcaagatggattaaagatttaaacgttagaccta 5800

||||||||||||||||||||||||||||||||||||||||||||||||||

**Canu_assem** 1732 TTATACAAAAATCAATTCAAGATGGATTAAAGATTTAAACGTTAGACCTA 1781

**LR_PCR_ref** 5801 aaaccataaaaaccctagaagaaaacctaggcattaccattcaggacata 5850

||||||||||||||||||||||||||||||||||||||||||||||||||

**Canu_assem** 1782 AAACCATAAAAACCCTAGAAGAAAACCTAGGCATTACCATTCAGGACATA 1831

**LR_PCR_ref** 5851 ggcgtgggcaaggacttcatgtccaaaacaccaaaagcaatggcaacaaa 5900

||||||||||||||||||||||||||||||||||||||||||||||||||

**Canu_assem** 1832 GGCGTGGGCAAGGACTTCATGTCCAAAACACCAAAAGCAATGGCAACAAA 1881

**LR_PCR_ref** 5901 agccaaaattgacaaatgggatctaattaaactcaagagcttctgcgcag 5950

||||||||||||||||||||||||||||||||||||||||||||||||||

**Canu_assem** 1882 AGCCAAAATTGACAAATGGGATCTAATTAAACTCAAGAGCTTCTGCGCAG 1931

**LR_PCR_ref** 5951 caaaagaaactaccatcagagtgaacaggcaacctacaacatgggagaaa 6000

||||||||||||||||||||||||||||||||||||||||||||||||||

**Canu_assem** 1932 CAAAAGAAACTACCATCAGAGTGAACAGGCAACCTACAACATGGGAGAAA 1981

**LR_PCR_ref** 6001 attttcgcaacctactcatctgacaaagggctaatatccagaatctacaa 6050

[5]

|||||||||||||||||||||||||||||||||||||.||||||||||||

**Canu_assem** 1982 ATTTTCGCAACCTACTCATCTGACAAAGGGCTAATATGCAGAATCTACAA 2031

[7]

[6]

**LR_PCR_ref** 6051 tgaactcaaacaaatttacaagaaaa---aaacaaccctatcaaaaagtg 6097

|||||||||||||||||||||||||| |||||||||.|||||||||||

**Canu_assem** 2032 TGAACTCAAACAAATTTACAAGAAAAAACAAACAACCCCATCAAAAAGTG 2081

**LR_PCR_ref** 6098 ggcgaaggacatgaacagacacttctcaaaagaagacatttatgcagcca 6147

||||||||||||||||||||||||||||||||||||||||||||||||||

**Canu_assem** 2082 GGCGAAGGACATGAACAGACACTTCTCAAAAGAAGACATTTATGCAGCCA 2131

**LR_PCR_ref** 6148 aaaaacacatgaagaaatgctcatcatcactggccatcagagaaatgcaa 6197

||||||||||||||||||||||||||||||||||||||||||||||||||

**Canu_assem** 2132 AAAAACACATGAAGAAATGCTCATCATCACTGGCCATCAGAGAAATGCAA 2181

**LR_PCR_ref** 6198 atcaaaaccactatgagatatcatctcacaccagttagaatggcaatcat 6247

||||||||||||||||||||||||||||||||||||||||||||||||||

**Canu_assem** 2182 ATCAAAACCACTATGAGATATCATCTCACACCAGTTAGAATGGCAATCAT 2231

**LR_PCR_ref** 6248 taaaaagtcaggaaacaacaggtgctggagaggatgtggagaaataggaa 6297

||||||||||||||||||||||||||||||||||||||||||||||||||

**Canu_assem** 2232 TAAAAAGTCAGGAAACAACAGGTGCTGGAGAGGATGTGGAGAAATAGGAA 2281

**LR_PCR_ref** 6298 cacttttacactgttggtgggactgtaaactagttcaaccattgtggaag 6347

||||||||||||||||||||||||||||||||||||||||||||||||||

**Canu_assem** 2282 CACTTTTACACTGTTGGTGGGACTGTAAACTAGTTCAACCATTGTGGAAG 2331

**LR_PCR_ref** 6348 tcagtgtagcgattcctcagggatctagaactagaaataccatttgaccc 6397

||||||||||||||||||||||||||||||||||||||||||||||||||

**Canu_assem** 2332 TCAGTGTAGCGATTCCTCAGGGATCTAGAACTAGAAATACCATTTGACCC 2381

**LR_PCR_ref** 6398 agccatcccattactgggtatatacccaaaggactataaatcatgctgct 6447

||||||||||||||||||||||||||||||||||||||||||||||||||

**Canu_assem** 2382 AGCCATCCCATTACTGGGTATATACCCAAAGGACTATAAATCATGCTGCT 2431

**LR_PCR_ref** 6448 ataaagacacatgcacacgtatgtttattgtggcactattcacaatagca 6497

||||||||||||||||||||||||||||||||||||||||||||||||||

**Canu_assem** 2432 ATAAAGACACATGCACACGTATGTTTATTGTGGCACTATTCACAATAGCA 2481

**LR_PCR_ref** 6498 aagacttggaaccaacccaaatgtccaacaatgatagactggattaagaa 6547

||||||||||||||||||||||||||||||||||||||||||||||||||

**Canu_assem** 2482 AAGACTTGGAACCAACCCAAATGTCCAACAATGATAGACTGGATTAAGAA 2531

**LR_PCR_ref** 6548 aatgtggcacatatacaccatggaatactatgcagccataaaaaatgatg 6597

||||||||||||||||||||||||||||||||||||||||||||||||||

**Canu_assem** 2532 AATGTGGCACATATACACCATGGAATACTATGCAGCCATAAAAAATGATG 2581

**LR_PCR_ref** 6598 agttcatgtcctttgtagggacatggatgaaattggaaaccatcattctc 6647

||||||||||||||||||||||||||||||||||||||||||||||||||

**Canu_assem** 2582 AGTTCATGTCCTTTGTAGGGACATGGATGAAATTGGAAACCATCATTCTC 2631

**LR_PCR_ref** 6648 agtaaactatcgcaagaacaaaaaaccaaacaccgcatattctcactcat 6697

||||||||||||||||||||||||||||||||||||||||||||||||||

**Canu_assem** 2632 AGTAAACTATCGCAAGAACAAAAAACCAAACACCGCATATTCTCACTCAT 2681

**LR_PCR_ref** 6698 aggtgggaattgaacaatgagatcacatggacacaggaaggggaatatca 6747

||||||||||||||||||||||||||||||||||||||||||||||||||

**Canu_assem** 2682 AGGTGGGAATTGAACAATGAGATCACATGGACACAGGAAGGGGAATATCA 2731

[9]

[8]

**LR_PCR_ref** 6748 cactctggggactgtggtggggtcgggggaggggggagggatagcattgg 6797

||||||||||||||.||||||||||||||| |||||||||||||||||||

**Canu_assem** 2732 CACTCTGGGGACTGCGGTGGGGTCGGGGGA-GGGGGAGGGATAGCATTGG 2780

**LR_PCR_ref** 6798 gagatatacctaatgctagatgacacgttagtgggtgcagcacaccagca 6847

||||||||||||||||||||||||||||||||||||||||||||||||||

**Canu_assem** 2781 GAGATATACCTAATGCTAGATGACACGTTAGTGGGTGCAGCACACCAGCA 2830

**LR_PCR_ref** 6848 tggcacatgtatacatatgtaactaacctgcacaatgtgcacatgtaccc 6897

||||||||||||||||||||||||||||||||||||||||||||||||||

**Canu_assem** 2831 TGGCACATGTATACATATGTAACTAACCTGCACAATGTGCACATGTACCC 2880

[10]

**LR_PCR_ref** 6898 taaaacttagagtataataaaaaatataaat------aaaaaaaaaaaaa 6941

||||||||||||||||||||||||||||||| |||||||||||||

**Canu_assem** 2881 TAAAACTTAGAGTATAATAAAAAATATAAATTAAAAAAAAAAAAAAAAAA 2930

**LR_PCR_ref** 6942 agaaaatctatttacttggatgggtttacagatttagttatcagctttcc 6991

||||||||||||||||||||||||||||||||||||||||||||||||||

**Canu_assem** 2931 AGAAAATCTATTTACTTGGATGGGTTTACAGATTTAGTTATCAGCTTTCC 2980

**LR_PCR_ref** 6992 tgactgttaggtatcttcttttgagaacaatttgagaaccagtttggtta 7041

||||||||||||||||||||||||||||||||||||||||||||||||||

**Canu_assem** 2981 TGACTGTTAGGTATCTTCTTTTGAGAACAATTTGAGAACCAGTTTGGTTA 3030

**LR_PCR_ref** 7042 tatgtttcaaaacacttttatattttttaaatagccaatctgctaaacaa 7091

||||||||||||||||||||||||||||||||||||||||||||||||||

**Canu_assem** 3031 TATGTTTCAAAACACTTTTATATTTTTTAAATAGCCAATCTGCTAAACAA 3080

**LR_PCR_ref** 7092 agcaggttactttaggttgagtacttttagtttgcagtttattggatgtc 7141

||||||||||||||||||||||||||||||||||||||||||||||||||

**Canu_assem** 3081 AGCAGGTTACTTTAGGTTGAGTACTTTTAGTTTGCAGTTTATTGGATGTC 3130

[11]

**LR_PCR_ref** 7142 ctgtaagttttgcttcctgtggatttttttccttttgcttgttatattaa 7191

||||||||||||||||||||||| ||||||||||||||||||||||||||

**Canu_assem** 3131 CTGTAAGTTTTGCTTCCTGTGGA-TTTTTTCCTTTTGCTTGTTATATTAA 3179

**LR_PCR_ref** 7192 atgtagattactgtcaattaagtctttagaggtccatccctaatcctgct 7241

||||||||||||||||||||||||||||||||||||||||||||||||||

**Canu_assem** 3180 ATGTAGATTACTGTCAATTAAGTCTTTAGAGGTCCATCCCTAATCCTGCT 3229

**LR_PCR_ref** 7242 ggcggcctctttaccacctcaccttgggcaggtctctatctgtacttcac 7291

||||||||||||||||||||||||||||||||||||||||||||||||||

**Canu_assem** 3230 GGCGGCCTCTTTACCACCTCACCTTGGGCAGGTCTCTATCTGTACTTCAC 3279

**LR_PCR_ref** 7292 aagggtgctgtggatcagggaaatgatgagtatgaagctgttttaaattc 7341

||||||||||||||||||||||||||||||||||||||||||||||||||

**Canu_assem** 3280 AAGGGTGCTGTGGATCAGGGAAATGATGAGTATGAAGCTGTTTTAAATTC 3329

**LR_PCR_ref** 7342 tcagatgaaaggttgtatgcaactacaaatcattatattatcttccacat 7391

||||||||||||||||||||||||||||||||||||||||||||||||||

**Canu_assem** 3330 TCAGATGAAAGGTTGTATGCAACTACAAATCATTATATTATCTTCCACAT 3379

**LR_PCR_ref** 7392 ccaaccacaagtgctctctagctttgaagtgcttcagttgactaattata 7441

||||||||||||||||||||||||||||||||||||||||||||||||||

**Canu_assem** 3380 CCAACCACAAGTGCTCTCTAGCTTTGAAGTGCTTCAGTTGACTAATTATA 3429

**LR_PCR_ref** 7442 tgttatcatgggctatttgaaactgactttatttgtgtgaagtaggaggc 7491

||||||||||||||||||||||||||||||||||||||||||||||||||

**Canu_assem** 3430 TGTTATCATGGGCTATTTGAAACTGACTTTATTTGTGTGAAGTAGGAGGC 3479

**LR_PCR_ref** 7492 agattagctagtatagttaatgtagtctcatctcagaaattatcagccca 7541

||||||||||||||||||||||||||||||||||||||||||||||||||

**Canu_assem** 3480 AGATTAGCTAGTATAGTTAATGTAGTCTCATCTCAGAAATTATCAGCCCA 3529

[12]

**LR_PCR_ref** 7542 tatggttgtacctaatgggcaagaaaagggggcatatgttggcctttcag 7591

||||||||||||||||||||||||||| ||||||||||||||||||||||

**Canu_assem** 3530 TATGGTTGTACCTAATGGGCAAGAAAA-GGGGCATATGTTGGCCTTTCAG 3578

**LR_PCR_ref** 7592 aaaatatttgcatggtatatttaattatttaagtagtacgtactcattat 7641

||||||||||||||||||||||||||||||||||||||||||||||||||

**Canu_assem** 3579 AAAATATTTGCATGGTATATTTAATTATTTAAGTAGTACGTACTCATTAT 3628

**LR_PCR_ref** 7642 aaaaatttcaaactctacagaaaaatatgaagtaagaaataattggcaat 7691

||||||||||||||||||||||||||||||||||||||||||||||||||

**Canu_assem** 3629 AAAAATTTCAAACTCTACAGAAAAATATGAAGTAAGAAATAATTGGCAAT 3678

**LR_PCR_ref** 7692 atgatacaaatgctctcatgtgtctctgtatcatcctttatttatttggt 7741

||||||||||||||||||||||||||||||||||||||||||||||||||

**Canu_assem** 3679 ATGATACAAATGCTCTCATGTGTCTCTGTATCATCCTTTATTTATTTGGT 3728

**LR_PCR_ref** 7742 gttcttgtagttggatatctgtgtactgatatctactacttgttctaagc 7791

||||||||||||||||||||||||||||||||||||||||||||||||||

**Canu_assem** 3729 GTTCTTGTAGTTGGATATCTGTGTACTGATATCTACTACTTGTTCTAAGC 3778

**LR_PCR_ref** 7792 tgctaagatgccacgtcatgtctatttaagaaaatttacattgttccatg 7841

||||||||||||||||||||||||||||||||||||||||||||||||||

**Canu_assem** 3779 TGCTAAGATGCCACGTCATGTCTATTTAAGAAAATTTACATTGTTCCATG 3828

[13]

**LR_PCR_ref** 7842 ccactatatgaaaatgtatacttaggtagttttttttaatagtgataatt 7891

||||||||||||||||||||||||||||| ||||||||||||||||||||

**Canu_assem** 3829 CCACTATATGAAAATGTATACTTAGGTAG-TTTTTTTAATAGTGATAATT 3877

**LR_PCR_ref** 7892 agtccatattatggtgataatgatggctacttgctgatccttagtgaaat 7941

||||||||||||||||||||||||||||||||||||||||||||||||||

**Canu_assem** 3878 AGTCCATATTATGGTGATAATGATGGCTACTTGCTGATCCTTAGTGAAAT 3927

**LR_PCR_ref** 7942 aaattctgtgttggtattcttcagcaaaaacgtcacattctgaacatcct 7991

||||||||||||||||||||||||||||||||||||||||||||||||||

**Canu_assem** 3928 AAATTCTGTGTTGGTATTCTTCAGCAAAAACGTCACATTCTGAACATCCT 3977

**LR_PCR_ref** 7992 aactaataaatcattgaccaggcattaggagagcatcttaacactctacc 8041

||||||||||||||||||||||||||||||||||||||||||||||||||

**Canu_assem** 3978 AACTAATAAATCATTGACCAGGCATTAGGAGAGCATCTTAACACTCTACC 4027

**LR_PCR_ref** 8042 cagtacatttaggtactctggttaatcaaatattatatgtagagtgatgg 8091

||||||||||||||||||||||||||||||||||||||||||||||||||

**Canu_assem** 4028 CAGTACATTTAGGTACTCTGGTTAATCAAATATTATATGTAGAGTGATGG 4077

[14]

**LR_PCR_ref** 8092 ccacttatcaaggaattggagggcaaaaaaatctctatattcactagttc 8141

|||||||||||||||||||||||| |||||||||||||||||||||||||

**Canu_assem** 4078 CCACTTATCAAGGAATTGGAGGGC-AAAAAATCTCTATATTCACTAGTTC 4126

**LR_PCR_ref** 8142 catgttcatgttttcaaatctttgtcgagtcatgtcaggctaatgatgtt 8191

||||||||||||||||||||||||||||||||||||||||||||||||||

**Canu_assem** 4127 CATGTTCATGTTTTCAAATCTTTGTCGAGTCATGTCAGGCTAATGATGTT 4176

**LR_PCR_ref** 8192 cttcagctttattataaagaacataaattatatggttcaaataaagacag 8241

||||||||||||||||||||||||||||||||||||||||||||||||||

**Canu_assem** 4177 CTTCAGCTTTATTATAAAGAACATAAATTATATGGTTCAAATAAAGACAG 4226

**LR_PCR_ref** 8242 actaataaagatttctgactctatgatatatgataaatagcctttactat 8291

||||||||||||||||||||||||||||||||||||||||||||||||||

**Canu_assem** 4227 ACTAATAAAGATTTCTGACTCTATGATATATGATAAATAGCCTTTACTAT 4276

**LR_PCR_ref** 8292 atcaatatagatgctaattagaattctgattattacttaatattctaagt 8341

||||||||||||||||||||||||||||||||||||||||||||||||||

**Canu_assem** 4277 ATCAATATAGATGCTAATTAGAATTCTGATTATTACTTAATATTCTAAGT 4326

**LR_PCR_ref** 8342 tttttccaaatataacttgaattttaaatgagaaaatatgaaagaaattt 8391

||||||||||||||||||||||||||||||||||||||||||||||||||

**Canu_assem** 4327 TTTTTCCAAATATAACTTGAATTTTAAATGAGAAAATATGAAAGAAATTT 4376

**LR_PCR_ref** 8392 gataacttacccattgatttatgaagaactaagtaggggtaaccttgaaa 8441

||||||||||||||||||||||||||||||||||||||||||||||||||

**Canu_assem** 4377 GATAACTTACCCATTGATTTATGAAGAACTAAGTAGGGGTAACCTTGAAA 4426

[15]

**LR_PCR_ref** 8442 cttgcctttgccctccctaaatatgggcaatggcagaatatgttcttgca 8491

|||||||||||||| |||||||||||||||||||||||||||||||||||

**Canu_assem** 4427 CTTGCCTTTGCCCT-CCTAAATATGGGCAATGGCAGAATATGTTCTTGCA 4475

**LR_PCR_ref** 8492 gacctataacttttgctttaaaactaagagactaggtgagtatatgatta 8541

||||||||||||||||||||||||||||||||||||||||||||||||||

**Canu_assem** 4476 GACCTATAACTTTTGCTTTAAAACTAAGAGACTAGGTGAGTATATGATTA 4525

**LR_PCR_ref** 8542 gacgggcactgttagaataattcccaaatgaatatagtttgtcagtggtt 8591

||||||||||||||||||||||||||||||||||||||||||||||||||

**Canu_assem** 4526 GACGGGCACTGTTAGAATAATTCCCAAATGAATATAGTTTGTCAGTGGTT 4575

**LR_PCR_ref** 8592 ctagggtagaggtaacctttaatttggtattcctaatagttcagaatgat 8641

||||||||||||||||||||||||||||||||||||||||||||||||||

**Canu_assem** 4576 CTAGGGTAGAGGTAACCTTTAATTTGGTATTCCTAATAGTTCAGAATGAT 4625

**LR_PCR_ref** 8642 gtatttatgctcatctctgcaaaattgtatatggttttttattactaatt 8691

||||||||||||||||||||||||||||||||||||||||||||||||||

**Canu_assem** 4626 GTATTTATGCTCATCTCTGCAAAATTGTATATGGTTTTTTATTACTAATT 4675

**LR_PCR_ref** 8692 ggtatttcatcttaacttgacagaatcttagtatcaattggtgaatcatt 8741

||||||||||||||||||||||||||||||||||||||||||||||||||

**Canu_assem** 4676 GGTATTTCATCTTAACTTGACAGAATCTTAGTATCAATTGGTGAATCATT 4725

**LR_PCR_ref** 8742 cggggtgagtattttctttctatgaaatataatagtatgcattgtaagta 8791

||||||||||||||||||||||||||||||||||||||||||||||||||

**Canu_assem** 4726 CGGGGTGAGTATTTTCTTTCTATGAAATATAATAGTATGCATTGTAAGTA 4775

**LR_PCR_ref** 8792 taaaagaaattaaagctttctataatttgaatttccaaatgcagttattc 8841

||||||||||||||||||||||||||||||||||||||||||||||||||

**Canu_assem** 4776 TAAAAGAAATTAAAGCTTTCTATAATTTGAATTTCCAAATGCAGTTATTC 4825

**LR_PCR_ref** 8842 aaacacctcatccaggcatattgcatagaattttatgagatatatatatc 8891

||||||||||||||||||||||||||||||||||||||||||||||||||

**Canu_assem** 4826 AAACACCTCATCCAGGCATATTGCATAGAATTTTATGAGATATATATATC 4875

**LR_PCR_ref** 8892 tcagatttactttcaaatcaagtttaatctcaaatcatactcctaattgg 8941

||||||||||||||||||||||||||||||||||||||||||||||||||

**Canu_assem** 4876 TCAGATTTACTTTCAAATCAAGTTTAATCTCAAATCATACTCCTAATTGG 4925

**LR_PCR_ref** 8942 tgaacttcaaaacttttctaaatatccacttgagattatataatacatat 8991

||||||||||||||||||||||||||||||||||||||||||||||||||

**Canu_assem** 4926 TGAACTTCAAAACTTTTCTAAATATCCACTTGAGATTATATAATACATAT 4975

**LR_PCR_ref** 8992 atacatttgtgtatatacatacatatatacgtgagctgtttttgctcaca 9041

||||||||||||||||||||||||||||||||||||||||||||||||||

**Canu_assem** 4976 ATACATTTGTGTATATACATACATATATACGTGAGCTGTTTTTGCTCACA 5025

[16]

**LR_PCR_ref** 9042 acatttctatcaccaaatgtgtgagatttttttctcacccaaatctattc 9091

|||||||||||||||||||||||||| |||||||||||||||||||||||

**Canu_assem** 5026 ACATTTCTATCACCAAATGTGTGAGA-TTTTTTCTCACCCAAATCTATTC 5074

**LR_PCR_ref** 9092 ttcaactctctggtgttctacaattcaattcaattctgacactaattacc 9141

||||||||||||||||||||||||||||||||||||||||||||||||||

**Canu_assem** 5075 TTCAACTCTCTGGTGTTCTACAATTCAATTCAATTCTGACACTAATTACC 5124

**LR_PCR_ref** 9142 cagagtcagcatcagactccacaggttcaagggctcagtcccacaaaaat 9191

||||||||||||||||||||||||||||||||||||||||||||||||||

**Canu_assem** 5125 CAGAGTCAGCATCAGACTCCACAGGTTCAAGGGCTCAGTCCCACAAAAAT 5174

**LR_PCR_ref** 9192 ggtctcactgcagacaccagtcacaagtgtcaggtccccaggctacacca 9241

||||||||||||||||||||||||||||||||||||||||||||||||||

**Canu_assem** 5175 GGTCTCACTGCAGACACCAGTCACAAGTGTCAGGTCCCCAGGCTACACCA 5224

[17]

**LR_PCR_ref** 9242 cacttccgtctgacttgaatacgaagttggggggttccgatagtgcctct 9291

|||||||||||||||||||||||||||| |||||||||||||||||||||

**Canu_assem** 5225 CACTTCCGTCTGACTTGAATACGAAGTT-GGGGGTTCCGATAGTGCCTCT 5273

**LR_PCR_ref** 9292 tccttacagtttgatccactgccagaactactcacaaaactctggaaaat 9341

||||||||||||||||||||||||||||||||||||||||||||||||||

**Canu_assem** 5274 TCCTTACAGTTTGATCCACTGCCAGAACTACTCACAAAACTCTGGAAAAT 5323

**LR_PCR_ref** 9342 attctacttactattatcagttcatcataaaagatacaaatgaacagcca 9391

||||||||||||||||||||||||||||||||||||||||||||||||||

**Canu_assem** 5324 ATTCTACTTACTATTATCAGTTCATCATAAAAGATACAAATGAACAGCCA 5373

**LR_PCR_ref** 9392 gatgaagaaatattatatagggtgaggtccagaagagtccctagcacagg 9441

||||||||||||||||||||||||||||||||||||||||||||||||||

**Canu_assem** 5374 GATGAAGAAATATTATATAGGGTGAGGTCCAGAAGAGTCCCTAGCACAGG 5423

**LR_PCR_ref** 9442 ggcttctgtccctggggagttggggtgcaccaccttcctagcacttagac 9491

||||||||||||||||||||||||||||||||||||||||||||||||||

**Canu_assem** 5424 GGCTTCTGTCCCTGGGGAGTTGGGGTGCACCACCTTCCTAGCACTTAGAC 5473

**LR_PCR_ref** 9492 atgtttaccaactccaaagatctcccaaccttattgttgaggggttttta 9541

||||||||||||||||||||||||||||||||||||||||||||||||||

**Canu_assem** 5474 ATGTTTACCAACTCCAAAGATCTCCCAACCTTATTGTTGAGGGGTTTTTA 5523

[18]

**LR_PCR_ref** 9542 tgggggtttcattatataggcataattgattaactcaatttccaaccccc 9591

||||||||||||||||||||||||||||||||||||||||||||| ||||

**Canu_assem** 5524 TGGGGGTTTCATTATATAGGCATAATTGATTAACTCAATTTCCAA-CCCC 5572

**LR_PCR_ref** 9592 tcccctccctggatagagggtggggctgaaagttccaagcttctactcaa 9641

||||||||||||||||||||||||||||||||||||||||||||||||||

**Canu_assem** 5573 TCCCCTCCCTGGATAGAGGGTGGGGCTGAAAGTTCCAAGCTTCTACTCAA 5622

**LR_PCR_ref** 9642 gacttggtctttctggcaaccagcttccatcctaaattagctaggtaccc 9691

||||||||||||||||||||||||||||||||||||||||||||||||||

**Canu_assem** 5623 GACTTGGTCTTTCTGGCAACCAGCTTCCATCCTAAATTAGCTAGGTACCC 5672

**LR_PCR_ref** 9692 accaagtatcacctcattagaacaaaagatggtcccatcacccttatcac 9741

||||||||||||||||||||||||||||||||||||||||||||||||||

**Canu_assem** 5673 ACCAAGTATCACCTCATTAGAACAAAAGATGGTCCCATCACCCTTATCAC 5722

**LR_PCR_ref** 9742 acatgaaattcgaagggttttaggagctctgtcccaggaaccagggacaa 9791

||||||||||||||||||||||||||||||||||||||||||||||||||

**Canu_assem** 5723 ACATGAAATTCGAAGGGTTTTAGGAGCTCTGTCCCAGGAACCAGGGACAA 5772

**LR_PCR_ref** 9792 agaccaaatatctttcaatgataccatgtatgtatgtacataacctcaca 9841

||||||||||||||||||||||||||||||||||||||||||||||||||

**Canu_assem** 5773 AGACCAAATATCTTTCAATGATACCATGTATGTATGTACATAACCTCACA 5822

[19]

**LR_PCR_ref** 9842 ggaatctttataaaacaattttgaaattcactcattatgagtgtgatttg 9891

|||||.||||||||||||||||||||||||||||||||||||||||||||

**Canu_assem** 5823 GGAATGTTTATAAAACAATTTTGAAATTCACTCATTATGAGTGTGATTTG 5872

**LR_PCR_ref** 9892 aaatgagatactccaaaatgtaagcccgatatccaaatgtcaccagcctg 9941

||||||||||||||||||||||||||||||||||||||||||||||||||

**Canu_assem** 5873 AAATGAGATACTCCAAAATGTAAGCCCGATATCCAAATGTCACCAGCCTG 5922

[20]

**LR_PCR_ref** 9942 tccctgcctactggtctccttccatacatatgcactttttgcttgtcctt 9991

||||||||||||||||||||||||||||||||||.|||||||||||||||

**Canu_assem** 5923 TCCCTGCCTACTGGTCTCCTTCCATACATATGCAGTTTTTGCTTGTCCTT 5972

**LR_PCR_ref** 9992 cctctcagacttctaggatattctttttctggtacactgattaggaattg 10041

||||||||||||||||||||||||||||||||||||||||||||||||||

**Canu_assem** 5973 CCTCTCAGACTTCTAGGATATTCTTTTTCTGGTACACTGATTAGGAATTG 6022

**LR_PCR_ref** 10042 tttgcatgagatcctgcctcagtgaaagtggcagagcttcattctaggag 10091

||||||||||||||||||||||||||||||||||||||||||||||||||

**Canu_assem** 6023 TTTGCATGAGATCCTGCCTCAGTGAAAGTGGCAGAGCTTCATTCTAGGAG 6072

**LR_PCR_ref** 10092 atccaagggaaagctttgctttgaaacatttattctaggctgcaaatcca 10141

||||||||||||||||||||||||||||||||||||||||||||||||||

**Canu_assem** 6073 ATCCAAGGGAAAGCTTTGCTTTGAAACATTTATTCTAGGCTGCAAATCCA 6122

**LR_PCR_ref** 10142 caaccctagttggccttccattaaagtcactaattcagcagtcccatatt 10191

||||||||||||||||||||||||||||||||||||||||||||||||||

**Canu_assem** 6123 CAACCCTAGTTGGCCTTCCATTAAAGTCACTAATTCAGCAGTCCCATATT 6172

**LR_PCR_ref** 10192 caatatgcattactgttaatatgttgcaccatctccattcccctgagagc 10241

||||||||||||||||||||||||||||||||||||||||||||||||||

**Canu_assem** 6173 CAATATGCATTACTGTTAATATGTTGCACCATCTCCATTCCCCTGAGAGC 6222

**LR_PCR_ref** 10242 ttatatttttaatttttaaatttttatttttagagacagtgtctcactct 10291

||||||||||||||||||||||||||||||||||||||||||||||||||

**Canu_assem** 6223 TTATATTTTTAATTTTTAAATTTTTATTTTTAGAGACAGTGTCTCACTCT 6272

**LR_PCR_ref** 10292 gtcacctacttattataacctcaaactcctcggcccaagcagtcctctca 10341

||||||||||||||||||||||||||||||||||||||||||||||||||

**Canu_assem** 6273 GTCACCTACTTATTATAACCTCAAACTCCTCGGCCCAAGCAGTCCTCTCA 6322

**LR_PCR_ref** 10342 ccttagcctcccaagttgccaggactacaggcatgcaccaccatgtccag 10391

||||||||||||||||||||||||||||||||||||||||||||||||||

**Canu_assem** 6323 CCTTAGCCTCCCAAGTTGCCAGGACTACAGGCATGCACCACCATGTCCAG 6372

**LR_PCR_ref** 10392 ctaatttttaaattttttgtagagacagggttttctatgttggccagatt 10441

||||||||||||||||||||||||||||||||||||||||||||||||||

**Canu_assem** 6373 CTAATTTTTAAATTTTTTGTAGAGACAGGGTTTTCTATGTTGGCCAGATT 6422

[21]

**LR_PCR_ref** 10442 ggtattgaactcctggcttccacgataccccgtctcagcctcccaaagaa 10491

|||||||||.||||||||||||||||||||||||||||||||||||||||

**Canu_assem** 6423 GGTATTGAATTCCTGGCTTCCACGATACCCCGTCTCAGCCTCCCAAAGAA 6472

**LR_PCR_ref** 10492 ctgggattacagatgtgagccactgcacctggccagagagcttatattct 10541

||||||||||||||||||||||||||||||||||||||||||||||||||

**Canu_assem** 6473 CTGGGATTACAGATGTGAGCCACTGCACCTGGCCAGAGAGCTTATATTCT 6522

**LR_PCR_ref** 10542 tataggaatgggaagactgcctatgttatgtgttgctacataatacatta 10591

||||||||||||||||||||||||||||||||||||||||||||||||||

**Canu_assem** 6523 TATAGGAATGGGAAGACTGCCTATGTTATGTGTTGCTACATAATACATTA 6572

**LR_PCR_ref** 10592 cccccaaacttagtgacttaaaacaaacgcttattatctccatttctgtg 10641

||||||||||||||||||||||||||||||||||||||||||||||||||

**Canu_assem** 6573 CCCCCAAACTTAGTGACTTAAAACAAACGCTTATTATCTCCATTTCTGTG 6622

**LR_PCR_ref** 10642 ggtcaataatctaggcatgacttagctgggccagagtttctccaaagtct 10691

||||||||||||||||||||||||||||||||||||||||||||||||||

**Canu_assem** 6623 GGTCAATAATCTAGGCATGACTTAGCTGGGCCAGAGTTTCTCCAAAGTCT 6672

[22]

**LR_PCR_ref** 10692 gtgatcaaggtgtcagttgggctgggcctgcagtcatctcaaggctccac 10741

||||||||||||||||| ||||||||||||||||||||||||||||

**Canu_assem** 6673 GTGATCAAGGTGTCAGT-----TGGGCCTGCAGTCATCTCAAGGCTCCAC 6717

**LR_PCR_ref** 10742 tagaggagcattcactggcagacttattcaaatggctgttggctgatcct 10791

||||||||||||||||||||||||||||||||||||||||||||||||||

**Canu_assem** 6718 TAGAGGAGCATTCACTGGCAGACTTATTCAAATGGCTGTTGGCTGATCCT 6767

[23]

**LR_PCR_ref** 10792 cgatggctattggcccctctattggtttcttgcccttgggcccctccata 10841

|||||||||||||||||||||||||||||||||||||||| |||||||||

**Canu_assem** 6768 CGATGGCTATTGGCCCCTCTATTGGTTTCTTGCCCTTGGG-CCCTCCATA 6816

**LR_PCR_ref** 10842 gtactgcttgctattcacaacatggcagcttgctttgcccagagcaggga 10891

||||||||||||||||||||||||||||||||||||||||||||||||||

**Canu_assem** 6817 GTACTGCTTGCTATTCACAACATGGCAGCTTGCTTTGCCCAGAGCAGGGA 6866

**LR_PCR_ref** 10892 ctctgagggaggcagggaaataaagagcaagagagaggtcacagtcttat 10941

||||||||||||||||||||||||||||||||||||||||||||||||||

**Canu_assem** 6867 CTCTGAGGGAGGCAGGGAAATAAAGAGCAAGAGAGAGGTCACAGTCTTAT 6916

**LR_PCR_ref** 10942 tgtaatctaattctggaaatgacagcccattacttttggcatattatttt 10991

||||||||||||||||||||||||||||||||||||||||||||||||||

**Canu_assem** 6917 TGTAATCTAATTCTGGAAATGACAGCCCATTACTTTTGGCATATTATTTT 6966

**LR_PCR_ref** 10992 ggttagaagcaagacaacagtagatctagcccacacacgaggggaggagg 11041

||||||||||||||||||||||||||||||||||||||||||||||||||

**Canu_assem** 6967 GGTTAGAAGCAAGACAACAGTAGATCTAGCCCACACACGAGGGGAGGAGG 7016

**LR_PCR_ref** 11042 atcacacaaggaggtgaataccaggaggtggggtcattgggagccatctg 11091

||||||||||||||||||||||||||||||||||||||||||||||||||

**Canu_assem** 7017 ATCACACAAGGAGGTGAATACCAGGAGGTGGGGTCATTGGGAGCCATCTG 7066

**LR_PCR_ref** 11092 agaggctgcccaccacactgcctcaagtaactagggagaggtaaaagttt 11141

||||||||||||||||||||||||||||||||||||||||||||||||||

**Canu_assem** 7067 AGAGGCTGCCCACCACACTGCCTCAAGTAACTAGGGAGAGGTAAAAGTTT 7116

**LR_PCR_ref** 11142 atatgccagatgaccaaatattaaaatgtgtgttacaaatagttcacgat 11191

||||||||||||||||||||||||||||||||||||||||||||||||||

**Canu_assem** 7117 ATATGCCAGATGACCAAATATTAAAATGTGTGTTACAAATAGTTCACGAT 7166

**LR_PCR_ref** 11192 gggctcagctgtcagactttacaaaggagctatgggaccttataaggaca 11241

||||||||||||||||||||||||||||||||||||||||||||||||||

**Canu_assem** 7167 GGGCTCAGCTGTCAGACTTTACAAAGGAGCTATGGGACCTTATAAGGACA 7216

**LR_PCR_ref** 11242 gttggaactggctaggtatcacatagtggtcttcaaacatttttgcttgc 11291

||||||||||||||||||||||||||||||||||||||||||||||||||

**Canu_assem** 7217 GTTGGAACTGGCTAGGTATCACATAGTGGTCTTCAAACATTTTTGCTTGC 7266

**LR_PCR_ref** 11292 cataacctctaaaataattgggaaaaagttgaatgtacttccatatctta 11341

||||||||||||||||||||||||||||||||||||||||||||||||||

**Canu_assem** 7267 CATAACCTCTAAAATAATTGGGAAAAAGTTGAATGTACTTCCATATCTTA 7316

**LR_PCR_ref** 11342 aagctgataatttaaaatattatacatttaatagcagcacgggatttagt 11391

||||||||||||||||||||||||||||||||||||||||||||||||||

**Canu_assem** 7317 AAGCTGATAATTTAAAATATTATACATTTAATAGCAGCACGGGATTTAGT 7366

**LR_PCR_ref** 11392 ttttgttaaattgtatatgtgctccaaatagatttaccatcaaaacctgt 11441

||||||||||||||||||||||||||||||||||||||||||||||||||

**Canu_assem** 7367 TTTTGTTAAATTGTATATGTGCTCCAAATAGATTTACCATCAAAACCTGT 7416

**LR_PCR_ref** 11442 tttgaatttaatattgggagaattcgctagtttaatttttggaaaataaa 11491

||||||||||||||||||||||||||||||||||||||||||||||||||

**Canu_assem** 7417 TTTGAATTTAATATTGGGAGAATTCGCTAGTTTAATTTTTGGAAAATAAA 7466

**LR_PCR_ref** 11492 gtataattggcaaagctaatcctcactgttgaatctatccgtcaaatcag 11541

||||||||||||||||||||||||||||||||||||||||||||||||||

**Canu_assem** 7467 GTATAATTGGCAAAGCTAATCCTCACTGTTGAATCTATCCGTCAAATCAG 7516

**LR_PCR_ref** 11542 atataatttctatcagaaagtctatatgacttgtcaacataatacccata 11591

||||||||||||||||||||||||||||||||||||||||||||||||||

**Canu_assem** 7517 ATATAATTTCTATCAGAAAGTCTATATGACTTGTCAACATAATACCCATA 7566

**LR_PCR_ref** 11592 aagtgaatcaaaaattattattcattgaacacatcatctcttatcaaatt 11641

||||||||||||||||||||||||||||||||||||||||||||||||||

**Canu_assem** 7567 AAGTGAATCAAAAATTATTATTCATTGAACACATCATCTCTTATCAAATT 7616

**LR_PCR_ref** 11642 cttgtgaccttccttctggttgtataatagcctaaaaaacaaaaaaagga 11691

||||||||||||||||||||||||||||||||||||||||||||||||||

**Canu_assem** 7617 CTTGTGACCTTCCTTCTGGTTGTATAATAGCCTAAAAAACAAAAAAAGGA 7666

**LR_PCR_ref** 11692 caaaagcaagtttccagaaagctgttctgacttgcctacttctgaaaagt 11741

||||||||||||||||||||||||||||||||||||||||||||||||||

**Canu_assem** 7667 CAAAAGCAAGTTTCCAGAAAGCTGTTCTGACTTGCCTACTTCTGAAAAGT 7716

**LR_PCR_ref** 11742 agtcctgtatggtgggttctgaaaATGAGGAACCAGGACTTGCA-- 11785

||||||||||||||||||||||||||||||||||||||||||||

**Canu_assem** 7717 AGTCCTGTATGGTGGGTTCTGAAAATGAGGAACCAGGACTTGCAAG 7762

#---------------------------------------

#---------------------------------------
