# Supplementary material for: Long‐read nanopore DNA sequencing can resolve complex intragenic duplication/deletion variants, providing information to enable preimplantation genetic diagnosis
Source: Prenat Diagn. 2022 Jan 18;42(2):226–32. doi: 10.1002/pd.6089 (PMC9305782; doi:10.1002/pd.6089)
Supplement: Supplementary file 3 — Figure S3 [file PD-42-226-s005.pdf]

Mismatch location	Mismatch defined following pairwise comparison of Canu assembled reads to the human reference genome (hg19)	Proportion of each nucleotide at SNV sites (determined from aligned nanopore reads)	Sanger sequencing validation	Final result
1	Deletion of 10 "T" nts from a 24 nt poly(T) tract			Sanger sequencing failed; variant unconfirmed
2	SNV	**Chr13:49,043,803**<br>T: 1208 (95%)<br>A: 18 (1%)<br>C: 39 (3%) Ref<br>G: 4 (0%)<br>Total: 1269 (100%)	Reference: TCTAGAAAACCCCATCGTCTCAGCCCAAAAT<br>Assembly: TCTAGAAAACCCCATTGTCTCAGCCCAAAAT<br>Sanger: TCTAGAAAACCCCATTGTCTCAGCCCAAAAT<br>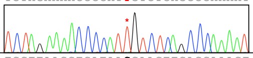	SNV confirmed
3	SNV	**Chr13:49,043,835**<br>T: 6 (0%)<br>A: 1193 (95.0%)<br>C: 4 (0%)<br>G: 54 (4%) Ref<br>Total: 1257 (100%)	Reference: TCCTTAAGCTGATAAGCAACTTCAGCAAAGT<br>Assembly: TCCTTAAGCTGATAAACAACTTCAGCAAAGT<br>Sanger: TCCTTAAGCTGATAAACAACTTCAGCAAAGT<br>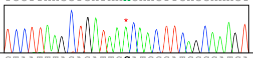	SNV confirmed
4	SNV	**Chr13:49,044,143**<br>T: 5 (0%)<br>A: 1211 (97%)<br>C: 8 (1%)<br>G: 23 (2%) Ref<br>Total: 1247 (100%)	Reference: GTAATTTACAGATTCGATGCCATCCCCATCA<br>Assembly: GTAATTTACAGATTCAATGCCATCCCCATCA<br>Sanger: GTAATTTACAGATTCAATGCCATCCCCATCA<br>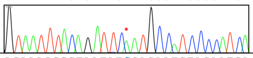	SNV confirmed
5	SNV	**Chr13:49,044,819**<br>T: 5 (0%)<br>A: 44 (4%)<br>C: 59 (5%) Ref<br>G: 1081 (91%)<br>Total: 1189 (100%)	Reference: ACAAAGGGCTAATATCCAGAATCTACAATGA<br>Assembly: ACAAAGGGCTAATATGCAGAATCTACAATGA<br>Sanger: ACAAAGGGCTAATATGCAGAATCTACAATGA<br>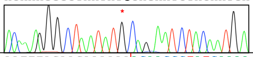	SNV confirmed
6	Insertion of "AAC" into a 7 nt poly(A) tract		Reference: AATTTACAAGAAAAAAACAACCCTATCAAAA<br>Assembly: AATTTACAAGAAAAAACAAACAACCCCATCA<br>Sanger: AATTTACAAGAAAAAAACAAACAACCCCATC<br>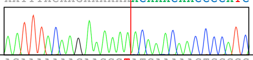	Poly(A) tract length undercalled by a single "A" nucleotide. Inserted non-ref "C" correctly identified.
7	SNV	**Chr13:49,044,867**<br>T: 189 (16%) Ref<br>A: 2 (0%)<br>C: 1020 (84%)<br>G: 8 (1%)<br>Total: 1219 (100%)	Reference: AGAAAAAAACAACCCTATCAAAAAGTGGGCG<br>Assembly: AAAAACAAACAACCCCATCAAAAAGTGGGCG<br>Sanger: AAAAACAAACAACCCCATCAAAAAGTGGGCG<br>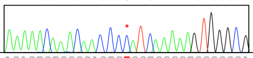	SNV confirmed
8	SNV	**Chr13:49,045,543**<br>T: 297 (25%) Ref<br>A: 30 (3%)<br>C: 818 (70%)<br>G: 30 (3%)<br>Total: 1175 (100%)	Reference: ACACTCTGGGGACTGTGGTGGGGTCGGGGGA<br>Assembly: ACACTCTGGGGACTGCGGTGGGGTCGGGGGA<br>Sanger: ACACTCTGGGGACTGCGGTGGGGTCGGGGGA<br>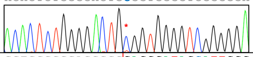	SNV confirmed
9	Deletion of a single "G" nt from a 6 nt poly(G) tract		Reference: GGTCGGGGGAGGGGGGAGGGATAGCATTGGG<br>Assembly: GGTCGGGGGAGGGGGAGGGATAGCATTGGGA<br>Sanger: GGTCGGGGGAGGGGGGAGGGATAGCATTGGG<br>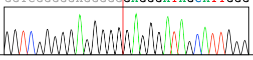	Poly(G) tract length undercalled by a single "G" nucleotide
10	Insertion of "TAAAAA" into a 14 nt poly(A) tract			Sanger sequencing failed; variant unconfirmed
11	Deletion of a single "T" nt from a 7 nt poly(T) tract		Reference: TTCCTGTGGATTTTTTTCCTTTTGCTTGTTA<br>Assembly: TTCCTGTGGATTTTTTCCTTTTGCTTGTTAT<br>Sanger: TTCCTGTGGATTTTTTTCCTTTTGCTTGTTA<br>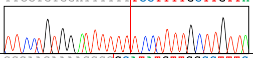	Poly(T) tract length undercalled by a single "T" nucleotide
12	Deletion of a single "G" nt from a 5 nt poly(G) tract		Reference: GGCAAGAAAAGGGGGCATATGTTGGCCTTTC<br>Assembly: GGCAAGAAAAGGGGCATATGTTGGCCTTTCA<br>Sanger: GGCAAGAAAAGGGGGCATATGTTGGCCTTTC<br>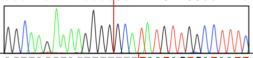	Poly(G) tract length undercalled by a single "G" nucleotide
13	Deletion of a single "T" nt from an 8 nt poly (T) tract		Reference: ACTTAGGTAGTTTTTTTTAATAGTGATAATT<br>Assembly: ACTTAGGTAGTTTTTTTAATAGTGATAATTA<br>Sanger: ACTTAGGTAGTTTTTTTTAATAGTGATAATT<br>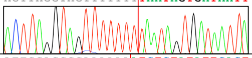	Poly(T) tract length undercalled by a single "T" nucleotide
14	Deletion of a single "A" nt from a 7 nt poly(A) tract		Reference: ATTGGAGGGCAAAAAAATCTCTATATTCACT<br>Assembly: ATTGGAGGGCAAAAAATCTCTATATTCACTA<br>Sanger: ATTGGAGGGCAAAAAAATCTCTATATTCACT<br>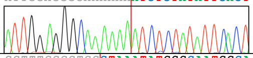	Poly(A) tract length undercalled by a single "A" nucleotide
15	Deletion of a single "C" nt from a 3 nt poly(C) tract		Reference: CCTTTGCCCTCCCTAAATATGGGCAATGGCA<br>Assembly: CCTTTGCCCTCCTAAATATGGGCAATGGCAG<br>Sanger: CCTTTGCCCTCCCTAAATATGGGCAATGGCA<br>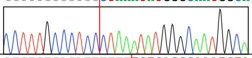	Poly(C) tract length undercalled by a single "C" nucleotide
16	Deletion of a single "T" nt from a 7 nt poly(T) tract		Reference: ATGTGTGAGATTTTTTTCTCACCCAAATCTA<br>Assembly: ATGTGTGAGATTTTTTCTCACCCAAATCTAT<br>Sanger: ATGTGTGAGATTTTTTTCTCACCCAAATCTA<br>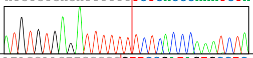	Poly(T) tract length undercalled by a single "T" nucleotide
17	Deletion of a single "G" nt from a 6 nt poly(G) tract		Reference: ATACGAAGTTGGGGGGTTCCGATAGTGCCTC<br>Assembly: ATACGAAGTTGGGGGTTCCGATAGTGCCTCT<br>Sanger: ATACGAAGTTGGGGGGTTCCGATAGTGCCTC<br>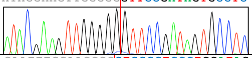	Poly(G) tract length undercalled by a single "G" nucleotide
18	Deletion of a single "C" nt from a 5 nt poly(C) tract		Reference: CAATTTCCAACCCCCTCCCCTCCCTGGATAG<br>Assembly: CAATTTCCAACCCCTCCCCTCCCTGGATAGA<br>Sanger: CAATTTCCAACCCCCTCCCCTCCCTGGATAG<br>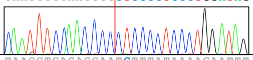	Poly(C) tract length undercalled by a single "C" nucleotide
19	SNV	**Chr13:49,048,628**<br>T: 39 (3%)<br>A: 24 (2%)<br>C: 58 (5%) Ref<br>G: 1146 (90%)<br>Total: 1267 (100%)	Reference: TAACCTCACAGGAATCTTTATAAAACAATTT<br>Assembly: TAACCTCACAGGAATGTTTATAAAACAATTT<br>Sanger: TAACCTCACAGGAATGTTTATAAAACAATTT<br>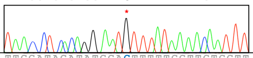	SNV confirmed
20	SNV	**Chr13:49,048,757**<br>T: 76 (8%)<br>A: 10 (1%)<br>C: 92 (9%) Ref<br>G: 828 (82%)<br>Total: 1006 (100%)	Reference: TTCCATACATATGCACTTTTTGCTTGTCCTT<br>Assembly: TTCCATACATATGCAGTTTTTGCTTGTCCTT<br>Sanger: TTCCATACATATGCAGTTTTTGCTTGTCCTT<br>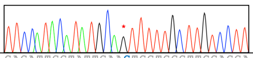	SNV confirmed
21	SNV	**Chr13:49,049,232**<br>T: 945 (83%)<br>A: 19 (2%)<br>C: 170 (15%) Ref<br>G: 8 (1%)<br>Total: 1142 (100%)	Reference: CAGATTGGTATTGAACTCCTGGCTTCCACGA<br>Assembly: CAGATTGGTATTGAATTCCTGGCTTCCACGA<br>Sanger: CAGATTGGTATTGAATTCCTGGCTTCCACGA<br>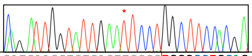	SNV confirmed
22	Deletion of "TGGGC"; not from within a poly(N) tract	**Chr13:49,049,490_49,049,494**<br>Deleted: 916 (71%)<br>Non-deleted: 381 (29%)	Reference: GATCAAGGTGTCAGTTGGGCTGGGCCTGCAG<br>Assembly: GATCAAGGTGTCAGTTGGGCCTGCAGTCATC<br>Sanger: GATCAAGGTGTCAGTTGGGCCTGCAGTCATC<br>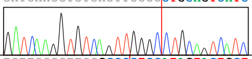	Deletion confirmed
23	Deletion of a single "C" nt from a 4 nt poly(C) tract		Reference: TCTTGCCCTTGGGCCCCTCCATAGTACTGCT<br>Assembly: TCTTGCCCTTGGGCCCTCCATAGTACTGCTT<br>Sanger: TCTTGCCCTTGGGCCCCTCCATAGTACTGCT<br>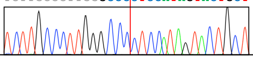	Poly(C) tract length undercalled by a single "C" nucleotide

Supplementary Figure 3: Sanger sequencing validation of mismatched nucleotides identified following pairwise alignment between the human reference sequence and Canu-generated consensus assembly. Mismatch locations correspond to the annotated sites in Supplementary Figure 2.
