# Supplementary material for: Long‐read nanopore DNA sequencing can resolve complex intragenic duplication/deletion variants, providing information to enable preimplantation genetic diagnosis
Source: Prenat Diagn. 2022 Jan 18;42(2):226–32. doi: 10.1002/pd.6089 (PMC9305782; doi:10.1002/pd.6089)
Supplement: Supplementary file 4 — Table S1 [file PD-42-226-s002.docx]

**Supplementary Table 1: Summary run metrics for long-read Flongle dataset**

| **Flowcell ID** | **Run yield** | **Reads generated** | **Selected read length (bp)** | **Quality filtering parameter** | **Median read length^** | **Total reads available for assembly^** | **Reads aligned within target region*** | **Mean per-base cumulative read depth^#^** |
| --- | --- | --- | --- | --- | --- | --- | --- | --- |
| ACM972 | 3.89 GB | 80,570 | 7500-8000 | 10 | 7,673 | 13,059 | 1,297 | 12,527× |

^Following adapter removal, read length filtering and quality score filtering. *Determined from downsampled BAM file which comprised 10% of the length and quality filtered reads (and excluded secondary or supplementary alignments). ^#^Determined from aligned BAM file, prior to subsampling. The target region was defined by the boundary of the LR-PCR primer sites (chr13:49038782-49050566).
