# Supplementary material for: Long‐read nanopore DNA sequencing can resolve complex intragenic duplication/deletion variants, providing information to enable preimplantation genetic diagnosis
Source: Prenat Diagn. 2022 Jan 18;42(2):226–32. doi: 10.1002/pd.6089 (PMC9305782; doi:10.1002/pd.6089)
Supplement: Supplementary file 5 — Table S2 [file PD-42-226-s001.docx]

**Supplementary Table 2: Pairwise identity, of the locus encompassing the variant site, between individual reads having the highest mean quality score and a curated reference sequence determined by Sanger sequencing.**

| **Read ID** | **Mean read quality (Q)** | **Total length (bp)** | **Pairwise identity (%)** |
| --- | --- | --- | --- |
| fcd3d9c1-f579-4897-996c-5c8d1fffd558 | 16.7 | 7,787 | 551/570 (96.7) |
| cba72790-49f8-43b9-ab4a-dd951ca7e0f5 | 16.3 | 7,803 | 560/568 (98.6) |
| f055f4c9-ebe5-4f4f-8777-698bcdc9daa2 | 16.2 | 7,712 | 546/568 (96.1) |
| 2f0750ec-32cf-4b2c-89b7-0c6c3a0636d4 | 16.1 | 7,795 | 549/568 (96.7) |
| 2a027d10-ee17-4ff3-b972-04233c544776 | 16.1 | 7,824 | 560/575 (97.4) |
